# Microcin PDI regulation and proteolytic cleavage are unique among known microcins

**DOI:** 10.1038/srep42529

**Published:** 2017-02-16

**Authors:** Zhe Zhao, Lisa H. Orfe, Jinxin Liu, Shao-Yeh Lu, Thomas E. Besser, Douglas R. Call

**Affiliations:** 1 College of Oceanography, Hohai University, Nanjing, 210098, P.R.China; 2 Paul G. Allen School for Global Animal Health, Washington State University, Pullman, WA, 99164, USA; 3 Department of Veterinary Microbiology and Pathology, Washington State University, Pullman, WA, 99164, USA

## Abstract

Microcin PDI inhibits a diversity of pathogenic *Escherichia coli* through the action of an effector protein, McpM. In this study we demonstrated that expression of the inhibitory phenotype is induced under low osmolarity conditions and expression is primarily controlled by the EnvZ/OmpR two-component regulatory system. Functional, mutagenesis and complementation experiments were used to empirically demonstrate that EnvZ is required for the inhibitory phenotype and that regulation of *mcpM* is dependent on binding of the phosphorylated OmpR to the *mcpM* promoter region. The phosphorylated OmpR may recognize three different binding sites within this promoter region. Site-directed mutagenesis revealed that the McpM precursor peptide includes two leader peptides that undergo sequential cleavage at positions G17/G18 and G35/A36 during export through the type I secretion system. Competition assays showed that both cleaved products are required for the PDI phenotype although we could not distinguish loss of function from loss of secretion in these assays. McpM has four cysteines within the mature peptide and site-directed mutagenesis experiments demonstrated that the first two cysteines are necessary for McpM to inhibit susceptible cells. Together these data combined with previous work indicate that MccPDI is unique amongst the microcins that have been described to date.

Competition between bacteria can be mediated by the presence of antibacterial compounds including classical antibiotics, lysozyme-like bacteriolytic enzymes, and bacteriocins^1^,^2^. Bacteriocins are a diverse group of peptides that are often only toxic to bacteria of the same species or genus as the producing strain^3^. Consequently, bacteriocins enhance the fitness of the producing strains and probably play an important role in structuring microbial communities. Bacteriocins offer the potential as alternatives to conventional antibiotics and for use in a variety of food safety applications^3^.

Bacteriocins produced by *Escherichia coli* are classified as either colicins or microcins based on their molecular mass^4^. Colicins are typically larger (>20 kDa) than microcins (<10 kDa)^5^,^6^. At this time, at least sixteen microcins have been identified. Microcins can be encoded on plasmids or chromosomes, usually with several genes that may be organized as an operon. These genes encode the precursor of the microcin, proteins needed for secretion, self-immunity factors and, in some situations, enzymes that are needed for post-translational modification of the microcins^6^. According to their structure and gene synteny, microcins are divided into two classes^7^. Class I microcins are small peptides (<5 kDa) that undergo extensive post-translational modifications^8^,^9^. Class II microcins are larger (5 to10 kDa) and are further subdivided into two subclasses, IIa and IIb. Class IIa includes plasmid-encoded peptides that do not undergo post-translational modification. Class IIb includes chromosomally encoded linear microcins that encode a C-terminal peptide sequence that signals a siderophore post-translational modification^10^.

Like bacteriocins from Gram-positive bacteria, microcins are generally derived from precursor peptides that are composed of a C-terminal core region and an N-terminal leader peptide. The leader peptide is typically cleaved during the process of export^6^,^11^. For example, the Colicin V precursor protein (a class IIa microcin) includes a double-glycine leader peptide consisting of 15 amino acid residues that is cleaved during export by the CvaA/CvaB/TolC export machinery^12^,^13^,^14^.

Microcin PDI (mccPDI) is a recently identified microcin that was first identified from a cattle-origin strain of *E. coli (E. coli*-25). MccPDI is of particular interest because it inhibits the growth of a broad diversity of *E. coli* including EHEC serotypes O157:H7 and O26^15^. The inhibitory phenotype has been called “proximity-dependent inhibition” (PDI) because inhibition only occurs when the microcin-producing cells are located in close proximity to sensitive cells^15^. Whole-genome sequence analysis identified five plasmid-encoded open-reading frames for mccPDI: *mcpM* and *mcpA* (microcin synthesis), *mcpI* (immunity) and *mcpD* and *mcpB* (export)^16^. The number and organization of the genes resembles that of the class IIa microcins. Gene deletions verified that these genes and chromosomally located *tol*C are responsible for the PDI phenotype. To date, however, gene regulation, protein maturation and protein function have not been determined for mccPDI. In this study we used the mccPDI-positive strain *E. coli*-25 (and genetic variants) and a strain of *E. coli* that is sensitive to mccPDI (strain BW25113 and genetic variants) to further dissect the process of transcriptional regulation and protein maturation. We determined that mccPDI expression is responsive to extracellular osmolarity, which is controlled by the EnvZ/OmpR two-component regulatory system. We further demonstrated that maturation of the mccPDI effector protein, McpM, involves two sequential proteolytic cleavage events that occur during the export process.

## Results

### MccPDI is regulated by the EnvZ/OmpR two-component regulatory system

**Figure 1:**
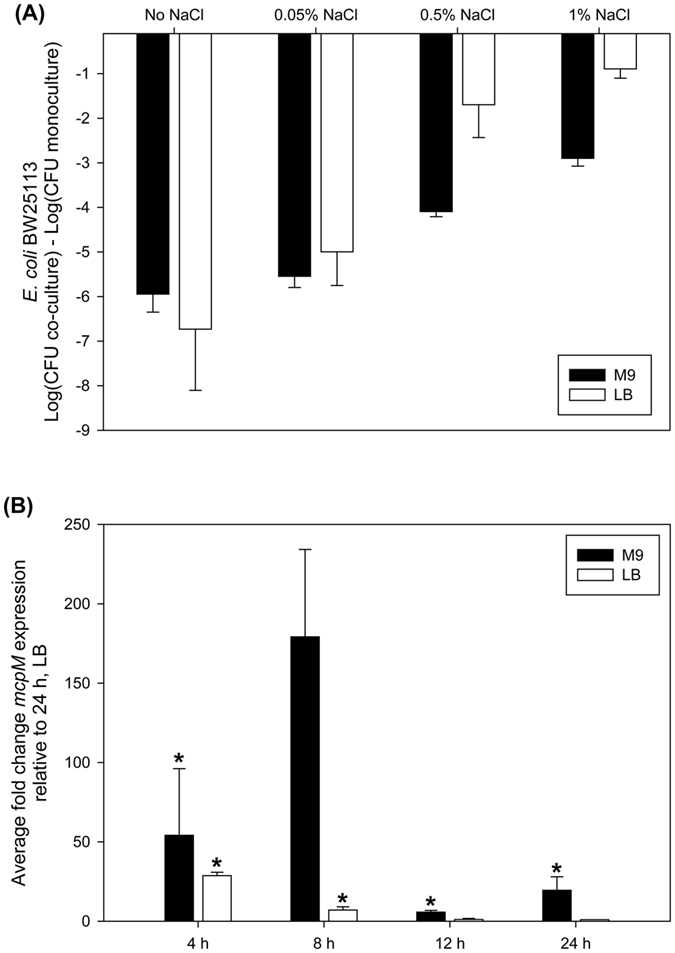
The mccPDI phenotype is linked to media osmolarity. (**A**) Competition assays between an mccPDI-producing strain (*E. coli*-25) and a target strain (BW25113) in M9 and LB with different concentrations of NaCl for 12 h. Results are expressed as the difference of mean log CFU during co-culture and mono-culture (n = 3 independent replicates; error bars = SEM). (**B**) Transcriptional analysis of *mcpM* for *E. coli*-25 cultured in LB or M9 media by qPCR. Fold change is expressed relative to *mcpM* expression in LB at 24 h (error bars = SEM; 3 independent replicates).*P < 0.01 based on ANOVA.

A previous study reported that inhibition by mccPDI was phenotypically obvious when strains were co-cultured in M9 medium (0.05% NaCl) but muted when co-cultured in LB medium (0.5% NaCl)^15^. Herein competition assays were performed between a mccPDI-producing strain (*E. coli*-25) and a susceptible strain (BW25113) in M9 and LB with different concentrations of NaCl. Resulting colony forming units (CFU; monoculture vs. co-culture) were quantified based on differential antibiotic selection (note that strain BW25113 is antibiotic sensitive, but we used a previously selected variant that is resistant to nalidixic acid)^17^. We found that the recovery of BW25113 was significantly lower in LB containing low (0.05% NaCl) or no added salt compared with media having higher salt concentrations (0.5% and 1%) (Fig. 1A). These results suggest that mccPDI regulation or function is sensitive to osmolarity. Consistent with altered expression, qPCR analysis revealed that transcription of *mcpM* was significantly upregulated at 8 h in lower-osmolarity M9 media when compared to higher osmolarity LB media (Fig. 1B). Transcription of other genes (*mcpI, mcpA,* and *mcpB*) within mccPDI gene cluster showed a similar pattern when cultured in LB and M9 media with the exception of *mcpB*, which was also upregulated at 24 h although not in LB (Fig. S1A).

**Figure 2:**
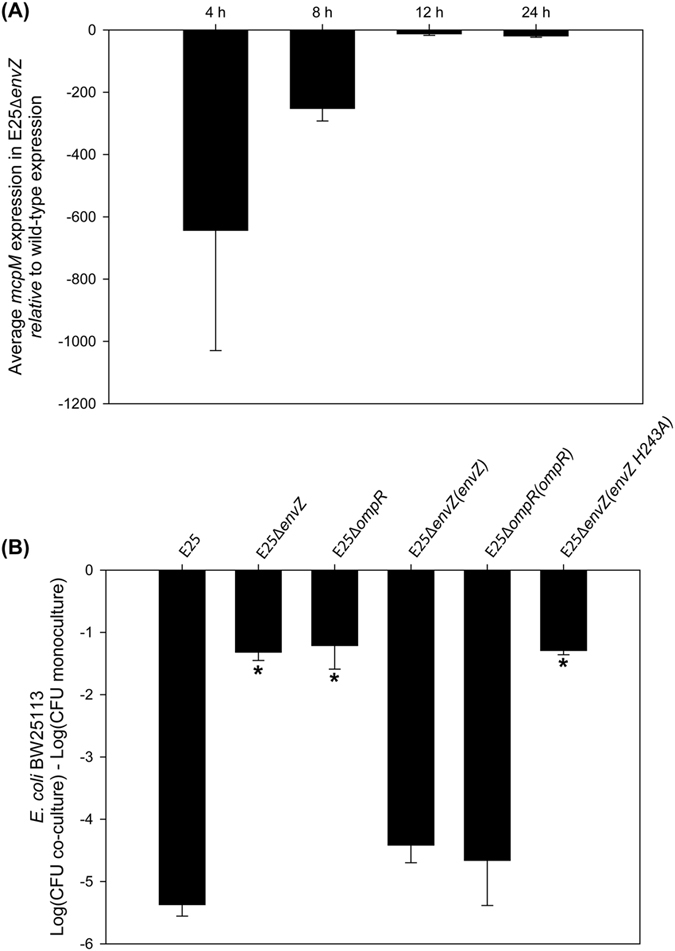
The EnvZ/OmpR two-component regulatory system controls the MccPDI phenotype. **(A**) Transcriptional analysis of *mcpM* for *E. coli*-25 and ∆*envZ* strains in M9 media (error bars = SEM; 2 independent replicates) by qPCR. *P* < 0.05 for all time points E25∆*envZ* versus WT at 8 h. GLM ANOVA followed by a Bonferroni multiple-comparison test. (**B**) ∆*envZ* or ∆*ompR* strains no longer exhibit the mccPDI phenotype. Results are shown for competition assays between different knockouts or their complemented strains and BW25113 or between an E25 vector control and BW25113. Results are expressed as the difference of BW25113 log CFUs during co-culture and mono-culture for 12 h (error bars = SEM; 3 independent replicates). For complementation experiments the competition assays were performed in M9 with 34 μg/ml chloramphenicol and 0.5 mM IPTG. *Significant ANOVA followed by a Dunnett’s one-way multiple comparisons test versus control group (E25) (*P* < 0.01).

Other authors have shown that the *E. coli* EnvZ/OmpR two-component system responds to osmolarity changes in broth media^18^,^19^. To determine if this system is involved with mccPDI regulation, *envZ* and *ompR* deletion strains (E25∆*envZ* and E25∆*ompR*) were constructed from the *E. coli*-25 parental strain (Table S1). When cultured in M9 media, the transcription of *mcpM* was down-regulated significantly, particularly at 4 h, for the E25∆*envZ* strain when compared to the isogenic control (Fig. 2A). Competition assays showed that the *envZ* or *ompR* gene knockout strains no longer inhibited the susceptible BW25113 strain while complementation of each gene restored the PDI phenotype (Fig. 2B). As further validation of these observations, we noted that Forst *et al*.^20^ previously demonstrated that a histidine residue at position 243 is critical to the function of EnvZ. We complemented our E25∆*envZ* strain with a version of *envZ* for which H243 was mutated to alanine and, as expected, this did not restore the inhibition phenotype (Fig. 2B). Collectively, these data are consistent with mccPDI being regulated by osmolarity that is signaled through the EnvZ/OmpR two-component system.

### Phosphorylated OmpR binds to the promoter region of *mcpM*

**Figure 3:**
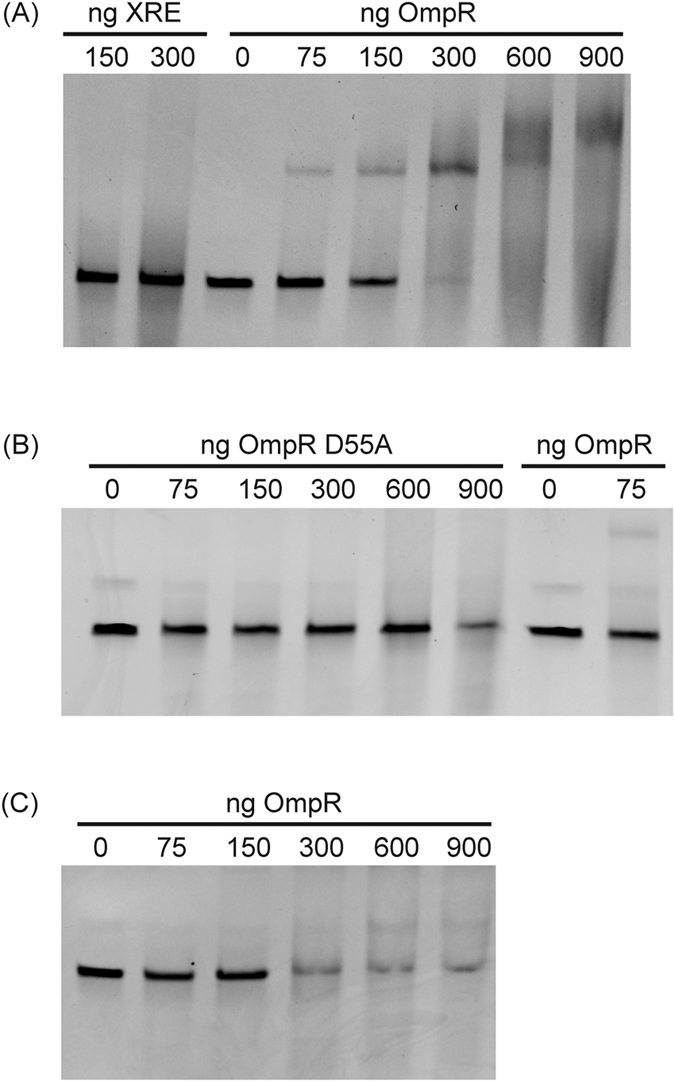
Phosphorylated OmpR binds to the promoter region of *mcpM*. (**A**) Electrophoretic mobility shift assays (EMSA) of a 200 bp DNA fragment located at position from −10 bp to −210 bp relative to the start codon of *mcpM* gene (P_mic-10/−210_). DNA (80 ng) was mixed with recombinant OmpR (0, 75, 150, 300, 600, 900 ng) or with an unrelated protein (XRE; Xenobiotic Response Element). (**B**) Unphosphorylated OmpR (Lane 2-Lane 6) for which the phosphorylation site (D55) was replaced by alanine and the protein no longer binds to P_mic-10/−210_. Normal OmpR (Lane 7 and 8) was included as control. (**C**) OmpR expressed and purified from BL21(DE3) in M9 broth (Lane 2- Lane 6) exhibited limited binding to P_mic-10/−210_.

EnvZ is an osmotic sensor that regulates the phosphorylation state of the transcriptional regulator, OmpR^21^. Phosphorylated OmpR controls the expression of outer membrane porin genes (*ompF* and *ompC*) and other virulence and fimbriae genes by binding their promoter region^19^,^22^,^23^. To determine if the OmpR binds to the promoter sequence of *mcpM*, a 200 bp DNA fragment located at position −10 bp to −210 bp (P_mic-10/−210_) relative to the start codon of *mcpM* was mixed with recombinant OmpR (Fig. S2) and this mixture was subjected to a mobility-shift assay. A concentration-dependent shift of the OmpR/DNA mixture was clearly evident and is consistent with increased mass associated with OmpR binding to the *mcpM* promoter region (Fig. 3A). Furthermore, when higher concentrations of OmpR (600 and 900 ng) were used in this assay, a more retarded band was observed, indicating the presence of larger mass products, which could arise if the promoter region of *mcpM* contains more than one OmpR-compatible binding site. The negative control protein, XRE (expressed and purified identically to recombinant OmpR; Fig. S2) did not bind the promoter of *mcpM* (Fig. 3A). Two negative control DNA fragments (270 bp, *atpE* and 201 bp, P_mic-233/−433_) displayed no gel shift after addition of purified OmpR (Fig. S3A). Sequence analysis showed that there is a non-coding region upstream from the ABC transporter genes (*mcpD* and *mcpB*) that could serve as another promoter sequence within the MccPDI gene cluster (Fig. S1B), but no gel shift was observed for this 143 bp DNA fragment (P_micD-20/−163_; relative to *mcpD* start codon) when mixed with recombinant OmpR (Fig. S3). Combined with the real-time data we can infer that *mcpM, mcpI* and *mcpA* are transcriptionally regulated as a unit by OmpR. The lack of OmpR binding proximal to *mcpD* and a different pattern of *mcpB* transcription relative to *mcpM, mcpI* and *mcpA* (Fig. S1A) suggests that *mcpD* and *mcpB* expression involves a different transcriptional regulator.

To determine if OmpR phosphorylation is required for binding to the *mcpM* promoter region, we mutated a conserved D55 residue of OmpR that is known to undergo transphosphorylation by EnvZ^18^. This mutated OmpR recombinant protein was mixed with P_mic-10/−210_ and the gel mobility shift assay showed no evidence of binding, even when 900 ng of protein was added (Fig. 3B). Consistent with this observation, OmpR purified from M9 media that contained a reduced amount of phosphorylated OmpR, displayed reduced binding to the *mcpM* promoter region when compared to the OmpR that was purified from LB media (Fig. 3C). Furthermore, OmpR did not bind P_mic-10/−210_ when the recombinant OmpR was dephosphorylated by treatment with calf intestinal alkaline phosphatase (Fig S3B). Taken together, this data indicates that OmpR binds to the promoter region of *mcpM* in a phosphorylation-dependent manner.

### *mcpM* promoter region includes more than one OmpR recognition site

**Figure 4:**
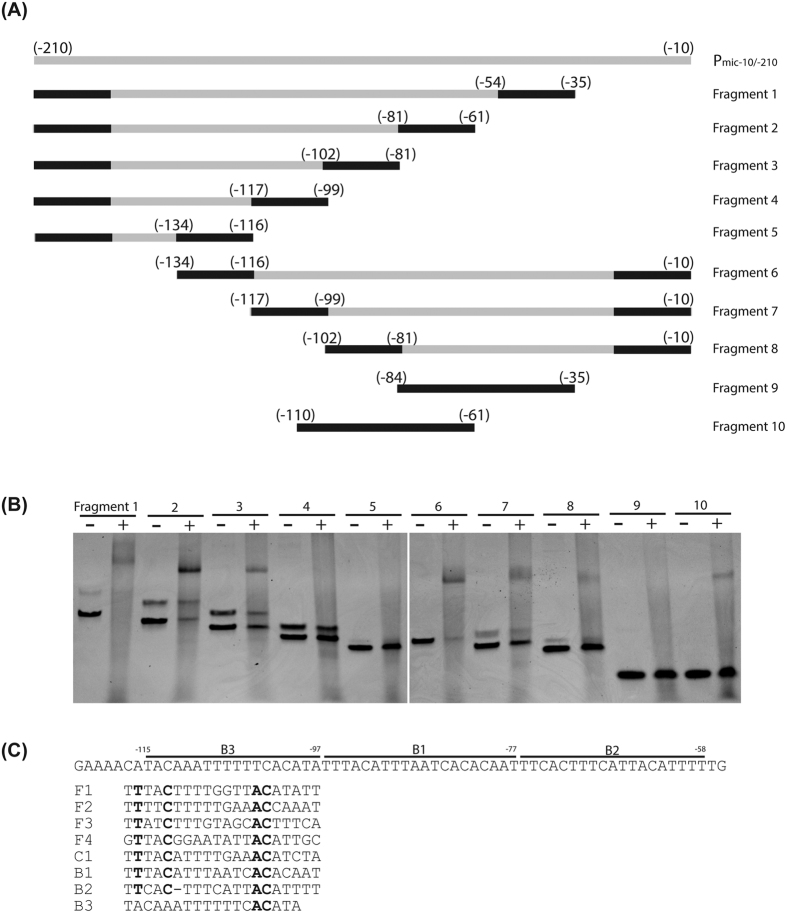
McpM promoter region has multiple binding sites for phosphorylated OmpR. (**A**) Diagram of the 10 DNA fragments that were selected from within the promoter region of *mcpM*. Fragments 1–8 were obtained by PCR amplification with black bars indicating the primer position. Fragments 9–10 were obtained by annealing complementary oligonucleotides. (**B**) EMSA of each DNA fragment (~80 ng) using 300 ng recombinant OmpR. Note that two PCR amplicons are present for several of the lanes that are attributable to mis-priming events in this AT-rich DNA sequence. (**C**) Three putative OmpR binding sites (B1, B2 and B3) are highlighted. F1, F2, F3 and F4 are the OmpR binding sites from the promoter region of *E. coli ompF* and C1 is the OmpR binding site identified with the promoter region of *E. coli ompC*.

To identify the sequence motif(s) that OmpR binds to within the P_mic-10/−210_ region, a series of truncated DNA fragments were prepared (Fig. 4A). OmpR bound DNA fragments 1–3, but not fragments 4 and 5 (Fig. 4B), indicating that the region between −81 to −102 is necessary for OmpR binding. Gel shift assays were consistent with binding for fragments 6–8 and fragment 10, which contain the region between −81 to −102, whereas fragment 9 did not bind to OmpR (Fig. 4B). From a qualitative perspective, binding was reduced for fragments 3, 7 and 8 compared with fragments 1, 2, and 6 (the same batch of recombinant OmpR was used for all of these mixtures). This could be a staining artifact (less ethidium bromide intercalated into shorter strands of DNA), but this is also consistent with sequences −61 to −81 and −102 to −134 providing additional binding sites. The *mcpM* promoter region is rich in adenines and thymines and three possible binding sites (B1, B2 and B3; Fig. 4C) are highlighted based on findings from Fig. 4B. A multiple sequence alignment showed that the three possible binding sites, especially B1, resemble the consensus OmpR binding sites for *ompF* and *ompC* (F1, F2, F3, F4 and C1) and include conserved nucleotides that are important for OmpR binding^24^. Collectively, these data are consistent with the *mcpM* promoter region having multiple binding sites and that region B1 is likely the primary binding site for the OmpR protein.

### McpM is cleaved into three peptides

**Figure 5:**
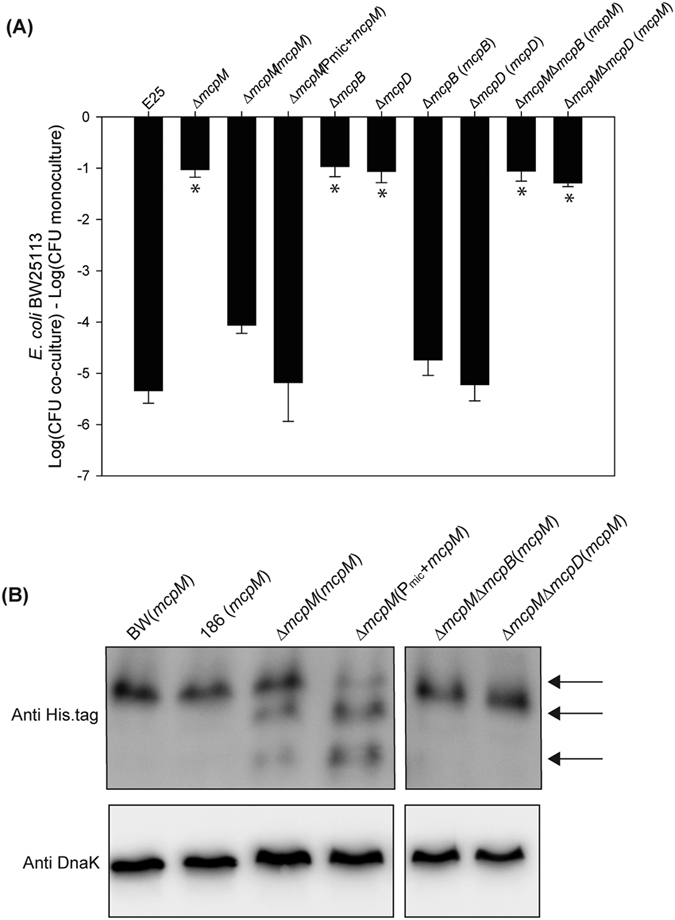
Competition results for MccPDI gene knockout and complementation experiments. (**A**) CFU counts for *E. coli* BW25113 following competition with microcin-producing *E. coli*-25 and associated gene knockout and complemented strains. Results are expressed as the difference in CFUs of the sensitive strain grown in co-culture and monoculture (error bars = SEM; 3 independent experiments). *Significant ANOVA followed by a Dunnett’s one-way multiple comparisons test versus control group (E25) (*P* < 0.01). (**B**) Western blot analysis of McpM in different *E. coli* strains, including BW25113, 186, and *mcpM*-complementation strains. Samples are from whole cell lysate and endogenous DnaK served as a loading control. Three bands, indicated by black arrows, correspond to the putative full length McpM (top) followed by two cleaved forms of the protein.

Eberhart *et al*.^16^ deleted the *mcpM* from *E. coli*-25, but complementation by *in trans* expression of *mcpM* was unsuccessful. The authors assumed this was due to a polar effect on the immunity gene, *mcpI*, owing to the proximal insertion of a kanamycin resistance gene in the knockout construct. For this reason, we generated a new scarless knock-out of *mcpM* using the suicide vector pDM4 in the wild-type *E. coli*-25 strain (Table S1). As expected, the inhibition phenotype was lost for the new knockout strain, but we were able to restore the phenotype upon *in trans* expression of *mcpM* driven by an IPTG-induced promoter (P*tac*) in the pMMB207 vector (Table S1 and Fig. 5A, lane 3). Please note that we refer to the native sequence as “wild type” for these experiments, although the recombinant form of the native sequence incorporates a C-terminal histidine tag for detection by western blot. Western blot analysis showed three distinct protein products in the *mcpM* complemented strains (Fig. 5B, lane 3). The upper band is the presumptive full-length protein because the estimated mass was consistent with the mass of the recombinant protein (also visible when synthesized in two MccPDI-susceptible strains, *E. coli* BW25113 and 186; Fig. 5B, lanes 1 and 2). These latter strains expressed recombinant McpM in the absence of other mccPDI-related proteins whereas the middle and lower bands (Fig. 5B, lane 3) from the *E. coli*-25 strain are probably cleaved forms of the full-length protein. Furthermore, cleavage is probably dependent on the presence of other mccPDI proteins. To exclude the alternative possibility that the promoter (P*tac*) affects the observed phenotype, we generated a recombinant *mcpM* construct with the native promoter (P_mic-1/−210_ + *mcpM*). The new construct displayed the same phenotype as the previous construct (p207::*mcpM*) that was driven by the P*tac* promoter (Fig. 5A,B, compare lanes 3 and 4).

### McpM undergoes two cleavage events

**Figure 6:**
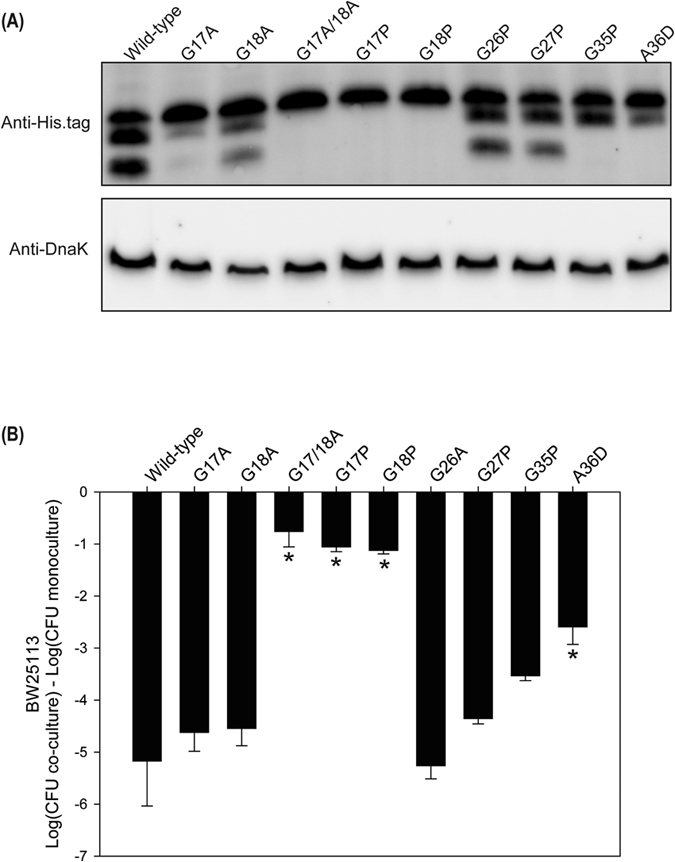
Identification of McpM cleavage sites. (**A**) Western blot analysis of McpM for the E25∆*mcpM*∆*mcpA* strain having different residue replacements in the McpM protein [designated (p207::*mcpM* XposY) in Table S1 where “X” is the wild-type amino acid, “pos” is the amino acid position, and “Y” is the replacement amino acid]. Samples are from cell lysate and endogenous DnaK served as a loading control. (**B**) Competition assays between the different site-specific mutants and BW25113 (vector ctrl). Results are expressed as the mean difference in CFU of the sensitive strain grown in co-culture and monoculture (error bars = SEM; 3 independent replicates). *Significant ANOVA followed by a Dunnett’s one-way multiple comparisons test versus control group (Wild-type) (*P* < 0.01).

Mature class II microcins are typically generated from proteolytic cleavage of a precursor protein that harbors conserved leader peptides^6^. Alignment of class IIa microcin precursors showed that a conserved double glycine is present in positions 17 and 18 of *mcpM*, consistent with the presence of an 18-residue leader peptide (Fig. S4A). When the glycine residues were changed to proline (G17P and G18P) this resulted in loss of two protein bands (Fig. 6A, lanes 5 and 6) relative to the wild-type strain (Fig. 6A, lane 1). Changing only one glycine to alanine (G17A or G18A) did not prevent cleavage although the faint-low mass protein bands are consistent with reduced cleavage efficiency for the G17A mutation (Fig. 6A, lanes 2 and 3). Double mutations from glycine to alanine (G17A/G18A) abolished cleavage (Fig. 6A, lane 4). Competition assays showed that these mutants are unable to inhibit sensitive strains (Fig. 6B, lanes 4–6).

To determine if McpM undergoes post-translational modification, we first submitted the middle protein band for peptide sequencing. This involved various protease treatments (trypsin, chymotrypsin, and elastase) followed by an analysis using UPLC-MS/MS with an Q-Exactive Orbitrap instrument. The results demonstrated no evidence for post-translational modifications (Bioproximity), which is consistent with the middle band being a product of cleavage rather than an otherwise modified form of the smaller protein (data not shown). To further explore this possibility, there is a second double glycine (positions 26 and 27) or a glycine-alanine motif at positions 35 and 36 could serve as a secondary cleavage sites for McpM (Fig. S4A). Mutations G26P and G27P had no effect on the western blot banding pattern (Fig. 6A, lanes 7 and 8) while mutations G35P and A36D resulted in loss of the lower-mass protein band (Fig. 6A, lanes 9 and 10). The most parsimonious conclusion from these observations is that the smaller protein band is generated from proteolytic cleavage of the middle band rather than the middle band being a post-translationally modified version of the smaller band. That is, McpM has two leader peptides (1–18 and 19–36) with the protein undergoing two cleavage events during maturation to produce the middle and smaller protein products. We further surmise that these events that are sequential rather than simultaneous.

A competition assay employing the mutated secondary cleavage site (G35P or A36D) displayed only partial inhibitory activity when compared with wild-type strain (Fig. 6B, bars 9 and 10 vs. bar 1). We also mutated other residues (R5, E11, V15, S16, N19, S33, R37 and G41) located within the two leader peptides and around the cleavage sites. Western blots showed that each mutant was processed normally (Fig. S4B), although E11A and V15A may have resulted in reduced synthesis of McpM. All mutants, except N19 exhibited comparable inhibition of a susceptible strain (Figure S4C).

### Cleavage of McpM is insufficient to produce a functional protein

**Figure 7:**
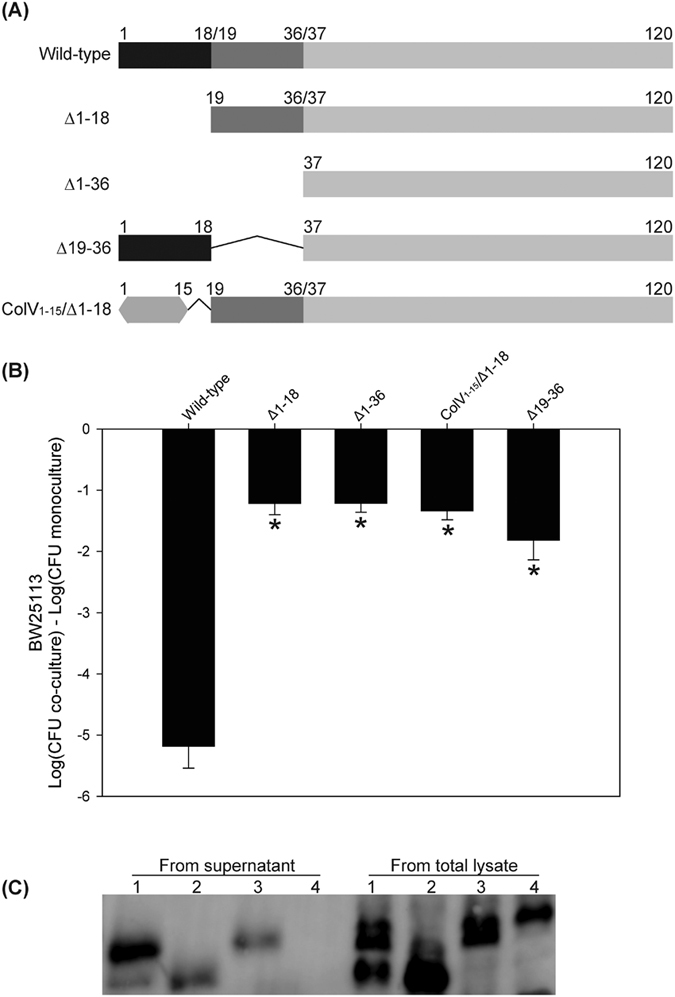
Loss of leader sequences blocks function of McpM. (**A**) Schematic diagram of different deleted constructs where residue numbering corresponds to amino acids in the full-length, wild-type McpM. ColV_1–15_ is the signal peptide sequence from colicin V. (**B**) Competition assays between the different deleted mutants and BW25113 (vector control). Deletion mutants were made in an E25∆*mcpM*∆*mcpA* and the modified recombinant protein was expressed *in trans* by using p207 (Table S1). Results are expressed as the mean difference in CFU of the sensitive strain grown in co-culture and monoculture (error bars = SEM; 3 independent replicates). *Significant ANOVA followed by a Dunnett’s one-way multiple comparisons test versus control group (Wild-type) (*P* < 0.01). (**C**) Western blot analysis of McpM that was precipitated from culture media. Lane 1: *E.coli*-25 (E25); Lane 2: E25∆*mcpM*∆*mcpA*(McpM∆19-36); Lane 3: E25∆*mcpM*∆*mcpA* (McpM A36D); Lane 4: E25∆*mcpM*∆*mcpB (mcpM*).

We generated different constructs of *mcpM* that lack individual leader peptides (∆1–18 or ∆19–36) or both (∆1–36; Fig. 7A). When these were expressed in the *E. coli*-25 background with a ∆*mcpM*∆*mcpA* mutation (Table S1), neither the ∆1–18 or ∆1–36 strains inhibited the sensitive BW25113 strain (Fig. 7B). Furthermore, western blots showed very limited quantities of McpM from these strains compared to the wild-type strain (Fig. S5). The Colicin V leader peptide shares ~50% amino acid identity with the primary leader sequence of McpM (Fig. S4A). Consequently, we replaced the primary leader peptide with the Colicin V leader peptide (ColV1–15/∆1–18) (Fig. 7A), but this was not sufficient to recover wild-type phenotype (Fig. 7B) and very limited quantities of McpM were again detected by western blot (Fig. S5).

Interestingly, the ∆19–36 strain appeared to have a similar concentration of protein as the wild-type strain (Fig. S5), but its inhibitory activity was significantly reduced (Fig. 7B). It is possible that the reduced activity is due to loss of export owing to the missing second leader sequence. Nevertheless, a western blot demonstrated that the product was present in TCA-precipitated culture supernatant (Fig. 7C). Both of the cleaved McpM products (but not the full-length protein) appeared to be present in the supernatant from *E. coli*-25 (positive control, lane 1) whereas no proteins were detected from the supernatant of the secretion-negative ∆*mcpB* strain (lane 4). While the ∆19–36 construct of McpM was exported (Fig. 7B, lane 2), it appears to have lost most of its functional activity (Fig. 7B, last bar). Alternatively, the molarity of secreted protein was too low to produce a wild-type phenotype. We did not have a means to normalize the amount of recombinant protein in these experiments.

### McpM cleavage is concomitant with export

Class II microcin export machinery displays a canonical structure consisting of three components. The ABC transporter and an accessory protein are encoded in the microcin gene cluster while the third component is the chromosome-encoded TolC^10^.We verified that ∆*mcpB* and ∆*mcpD* strains lose the inhibitory phenotype against stain BW2513 while complementation restores the phenotype (Fig. 5A, compare bars 5–6 with 7–8). Sequence alignment shows that McpB contains three conserved domains including an N-terminal peptidase C39 domain, an ABC transporter transmembrane domain, and a C-terminal ABC transporter ATP-binding domain (data not shown), which is consistent with the ABC transporter family. *In trans* expression of *mcpM* in the *E. coli*-25 background with a ∆*mcpM*∆*mcpB* deletion demonstrated that deletion of *mcpB* leads to the loss of McpM cleavage, consistent with the ABC transporter being responsible for cleavage of this protein (Fig. 5B, lane 5). McpD is homologous to proteins of class II microcin export machinery and it likely serves as a connector between the ABC transporter and TolC^12^,^25^. *In trans* expression of *mcpM* in a ∆*mcpM*∆*mcpD* strain did not affect production of full-length McpM, but cleavage was not observed as with the ∆*mcpB* strain (Fig. 5B, lane 6), consistent with cleavage of McpM being concomitant with export.

### The first two cysteines within the McpM are necessary for function

**Figure 8:**
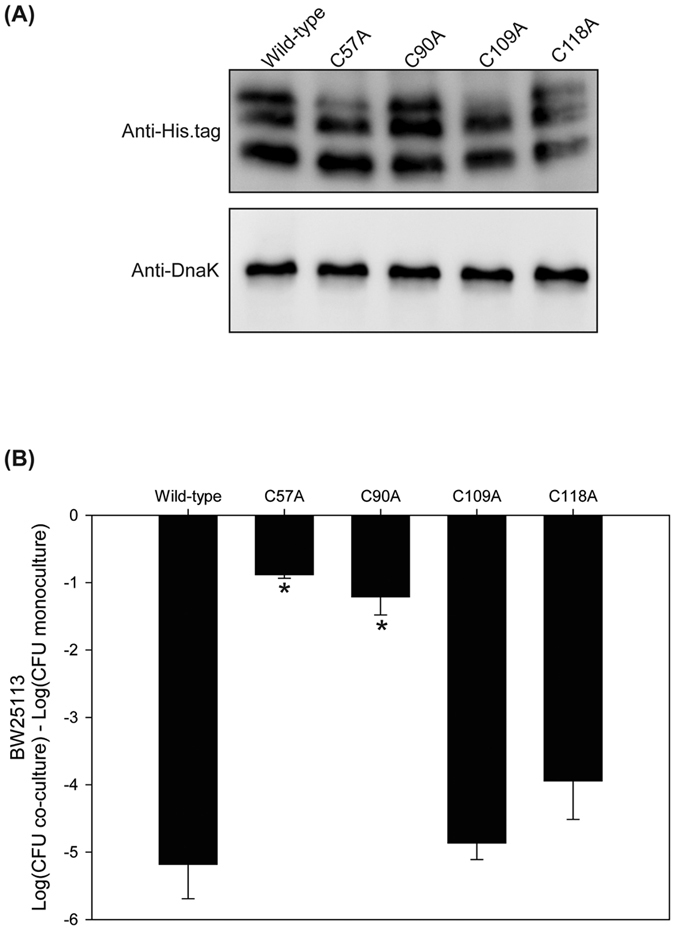
Cysteine residues 57 and 90 are required for McpM function. (**A**) Western blot analysis of McpM for strains (E25∆*mcpM*∆*mcpA*) with replacement of different cysteine residues protein [designated (p207::*mcpM* XposY) in Table S1 where “X” is the wild-type amino acid, “pos” is the amino acid position, and “Y” is the replacement amino acid]. Endogenous DnaK served as a loading control. (**B**) Competition assays between the 4 cysteine-residue mutants and BW25113 (vector control). Results are expressed as the difference in CFU of the sensitive strain grown in co-culture and monoculture (bars = SEM; 3 independent replicates). *Statistically significant ANOVA (*P* < 0.01 with Dunnett’s upper one-sided multiple-comparison test with control).

In class IIa microcins, cysteine pairs are commonly associated with the formation of disulfide bonds^6^. The McpM protein includes four cysteine residues (positions 57, 90, 109 and 118) and all are located within the mature peptide sequence (Fig. S4A). To determine if these cysteine residues are important to protein function, each was individually mutated to alanine using site-directed mutagenesis. Western blots demonstrated that these point mutations did not affect McpM synthesis or post-translational cleavage (Fig. 8A), but the PDI inhibitory phenotype was eliminated for the C57A and C90A mutations, whereas mutation of cysteines 3 and 4 (C109A and C118A) had no effect on function (Fig. 8B). These results are consistent with the possibility that a disulfide bond is required between the cysteine residues located at positions 57 and 90 for McpM to be functional. DsbA and DsbB are thiol-redox enzymes that are responsible for disulfide-bond formation in *E. coli*^26^. Knockouts of *dsbA* and *dsbB* (∆*dsbA* and ∆*dsbB*) in the *E. coli*-25 strain did not result in loss of PDI phenotype (Fig. S6). Furthermore, a series of double-knockouts (∆*dsbA*∆*dsb*B and ∆*dsbA*∆*dsbD*) or triple-knockout (∆*dsbA*∆*dsbB*∆*dsbD*) eliminated the possible redundancy between the DsbA/DsbB and DsbC/DsbD pathways, but did not impact the PDI phenotype (Fig. S6).

## Discussion

Bacteriocin production is an inducible process that is affected by different environmental and nutritional factors^27^. For example, expression of colicin genes is regulated by the SOS response regulon that responds to DNA damage^28^,^29^. Alternatively, regulation of microcin synthesis is more related to nutrient depletion or anoxic conditions^6^. For example, production of some class I microcins (MccB17, MccC7/C51 and MccJ25) is upregulated when cells reach the stationary growth phase^30^. One notable exception is MccE492, which is only produced during the exponential growth phase^31^. Nitrogen starvation induces MccB17 production^32^, and MccV production is initiated under iron-limiting conditions^12^.

MccPDI gene expression increases rapidly during log-phase growth and drops off as the population enters stationary phase^16^. The PDI phenotype is enhanced significantly when these experiments are conducted in M9 media compared to LB media, arguing that differences between the media (e.g., salt concentrations) might affect microcin synthesis or function^15^. We demonstrated that osmolarity in the growth media is a key signal for expression of the *mcpM*. This is a novel regulatory mechanism with respect to what is known about microcins, although osmolarity can influence bacteriocin production in Gram-positive bacteria^33^.

The EnvZ/OmpR two-component regulatory system plays a central role in mediating the response to osmotic stress in *E. coli*^34^. It was therefore not surprising to find that osmolarity-sensitive expression of mccPDI is dependent on the EnvZ/OmpR system where the phosphorylated transcriptional regulator, OmpR, binds to the *mcpM* promoter region. Similarly, Hernández-Chico *et al*.^35^ reported that expression of the MccB17 gene cluster is dependent on the OmpR transcriptional factor, but this regulation is growth-phase dependent.

The EnvZ/OmpR system also regulates synthesis of the outer membrane proteins OmpF and OmpC that enable bacteria to cope with fluctuations in osmolarity^20^. Under high osmolarity conditions, EnvZ auto-phosphorylates and transfers the phosphoryl group to OmpR, producing the phosphorylated form OmpR-P. At low osmolarity, OmpR-P is present in low concentrations. OmpR-P binds to the promoter regions of outer membrane porin genes *ompF* and *ompC* and differentially modulates their expression according to the concentration of cellular OmpR-P^36^. There are several binding sites for OmpR-P within the promoter region of *ompF*. When present in low concentrations, OmpR-P only binds to the high-affinity sites. Under high osmolarity conditions, OmpR-P concentration increases and binding occurs at low-affinity sites that result in reduced expression of *ompF*^24^. Here, we show that regulation of *mcpM* is negatively correlated with osmolarity of the growth media (greater in M9 than LB). OmpR-P clearly binds the promoter region of *mcpM* whereas unphosphorylated OmpR does not. Consequently, the EnvZ/OmpR system is required for activation of MccPDI and we propose that *mcpM* transcriptional regulation mirrors that of *ompF* regulation. This conclusion is further supported by the finding of at least three putative binding sites in the McpM promoter region that resemble the consensus OmpR binding site for *ompF*. Furthermore, Zhao *et al*.^17^ recently demonstrated that McpM interacts with OmpF of mccPDI susceptible strains and consequently, the concurrent expression of these traits in producer and susceptible cells likely maximizes the ability of the mccPDI-producing strains to efficiently inhibit susceptible competitors.

Functional microcins are usually derived from a precursor protein that is composed of a C-terminal structural region and an N-terminal leader peptide^11^. Enzymatic cleavage removes the leader peptide and the microcin may or may not undergo further post-translational modification. The class II microcins have conserved leader peptides that range in size from 15 to 19 residues and harbor a double-glycine or glycine-alanine cleavage site^6^. In contrast, there is little sequence similarity between the leader peptides of class I microcins. For example, the MccB17 precursor, a class I microcin, is processed at G_26_, but this cleavage site is not a typical sequence of the double-glycine-type leader peptides as described for class II microcins^37^. MccPDI most closely resembles a class IIa microcin based on its genetic organization^16^. The immature microcin protein, McpM, contains a typical double-glycine cleavage site (G_17_G_18_) and a conserved leader peptide (residues 1–18) similar to other class II microcins. McpM also harbors a second cleavage site (G_35_A_36_) corresponding to a second leader peptide sequence. To our knowledge, this is the first report of a microcin composed of two leader peptides (1–18 and 19–36).

Leader peptides typically prevent microcin function (e.g., in the cytoplasm of the producing strain) or serve as a recognition site for export^27^. For McpM, experimental evidence suggests that the first leader peptide, but not the second, is required for export (Fig. 7C). The first leader sequence may also serve to inhibit protein degradation because when absent we find only very small quantities of the processed McpM protein in the cell. We assume that the second cleavage event takes place during or after export. If the latter, this would be consistent with the hypothesis that the fully functional microcin is composed of a dimer or mulitimer of the two cleaved products (see below).

Cysteine is the least abundant amino acid found in proteins^38^ and it performs a variety of essential functions including binding metal ions and forming disulfide bonds that produce three-dimensional protein structures^39^. For these reasons, if a protein contains an “even” number of cysteines and is predicted to function outside the cytoplasm, it is likely that these cysteines form disulfide bonds^40^. For class IIa microcins, cysteines commonly form disulfide bonds in the mature peptide. The full-length MccB17 protein has four cysteine residues that form heterocyclic rings by an unusual post-translational modification of the mature microcin^41^, and mutational analysis suggests that the mature form of MccV has a disulfide bond between the cysteine residues at positions 76 and 87^42^. In addition, using mass spectrometry Pons *et al*.^25^ detected the presence of two intramolecular disulfide bridges in the mature MccL.

McpM has four cysteines within the mature protein, consistent with the prediction that disulfide-bond formation occurs. Our experimental data shows that the first two cysteines are necessary for mccPDI inhibition. We also conducted competition assays in the presence of 5 mM DTT, a reducing agent that breaks disulfide bonds. Under these conditions, no inhibition was observed (data not shown) although this type of experiment could have multiple confounding effects. There was no evidence in this study that Dsb-based enzymatic activity in the McpM-producing strain contributes to disulfide-bond formation, but other work shows that strains lacking DsbA or DsbB are less susceptible to mccPDI^17^. While a computational three-dimensional model for McpM did not support the formation of an intra-molecular disulfide bond between cysteine 57 and cysteine 90 due to the physical distance between these sites (data not shown), the reduced killing activity observed when only one of the two cleaved forms is present suggests the possibility of inter-molecular disulfide-bond formation. If disulfide bonds are required for function, we surmise that they form after the mature McpM protein enters the susceptible cell where folding likely occurs in the periplasm.

After maturation and export, microcins inhibit susceptible bacteria through a variety of mechanisms. MccJ25 recognizes the outer membrane protein FhuA and requires the inner membrane proteins TonB, ExbB, ExbD and SbmA, for translocation^43^,^44^,^45^. Once it reaches the cytoplasm, MccJ25 inhibits transcription by obstructing the RNA polymerase secondary channel^46^. MccB17 binds OmpF on the outer membrane and the inner protein SbmA mediates uptake into the cytoplasm, where MccB17 inhibits the DNA gyrase^47^. Microcin C7/C51 requires OmpF and the inner-membrane ABC-transporter, Yej, to be actively transported through the inner membrane^48^. Within the target cell MccC7/C51 is cleaved to form a modified aspartyl adenylate that inhibits Asp-tRNA synthetase, thus blocking protein synthesis at the translation level^49^,^50^. MccE492, MccM, and MccH47, all class IIb microcins, are unable to inhibit the growth of strains carrying mutations in the *fepA, cir*, and *fiu* genes, consistent with the requirement for these iron-catecholate receptors^51^,^52^.

The transport of class IIb microcins across the outer membrane is also TonB-dependent^51^,^53^. Once in the periplasm, MccE492 functions by inserting into the inner membrane and interfering with membrane potential^54^,^55^. This activity is facilitated by the inner membrane proteins ManY and ManZ^56^. MccH47 exerts its activity by inhibiting the ATP synthase^57^. MccV causes channel formation and disruption of membrane potential by binding to the inner membrane receptor SdaC^58^,^59^. Recently, it was shown that outer-membrane OmpF is necessary for mccPDI to harm susceptible *E. coli*^17^. We propose the following model for MccPDI function. First, McpM precursor protein undergoes two cleavage events to produce two cleaved forms during and possibly after export. The two cleaved peptides interact with OmpF of susceptible cells, crosses the outer membrane using an unknown mechanism to access the periplasm where disulfide bridges facilitate the formation of McpM multimers. The disulfide bonds are formed utilizing the target cell thio-redox systems, DsbA/B and/or DsbC/D. Preliminary data suggests that the multimer McpM proteins then permeabilize the susceptible-cell’s membrane leading to cell death.

## Methods

### Bacterial strains, media and growth conditions

*E. coli* strains (Table S1) were cultured in LB-Lennox medium (LB broth) (Difco) or in M9 minimal medium (6 g/L Na_2_HPO_4_, 3 g/L KH_2_PO_4_, 0.5 g/L NaCl, 1 g/L NH_4_Cl, 2 mg/L thiamine, 1 mM MgSO_4_, 0.1 mM CaCl_2_, and 0.2% glucose) at 37 °C with shaking (200 rpm). *E. coli*-25 (E25) is a mccPDI-producing strain that has been associated with resistance to the antibiotics streptomycin, sulfadiazine, and tetracycline (SSuT^r^)^15^. *E. coli* BW25113 is a mccPDI-susceptible strain. To distinguish BW25113 from *E. coli*-25 in a mixed culture, we used a previously selected variant of this strain that is resistant to nalidixic acid^17^. Unless otherwise indicated, antibiotics were added to media at the following concentrations: tetracycline (Tet), 50 μg/ml; chloramphenicol (Cm), 34 μg/ml; kanamycin (Kan), 50 μg/ml; nalidixic acid (Nal), 30 μg/ml; and ampicillin (Amp), 100 μg/ml. LB broth with different salt concentrations were made by mixing 10 g/L Bacto-tryptone, 5 g/L yeast extract and NaCl at indicated concentrations.

### DNA manipulation and mutant construction

Extraction of *E. coli* genomic DNA was accomplished using a DNeasy Blood & Tissue kit following the manufacturer’s instruction (Qiagen). Plasmid DNA was purified using a QIAprep Spin Miniprep Kit (Qiagen). Primer pairs (Eurofins Genomics) and corresponding restriction enzyme sequences (New England Biolabs) are detailed in Table S2. Conventional PCR included DreamTaq Green PCR Master Mix (Thermo Scientific) while preparative PCR used for plasmid construction was generated using Platinum PCR SuperMix High Fidelity (Invitrogen) according to the manufacturer’s protocol.

Deletion cassettes for chromosomal in-frame deletions were first generated using the splice-overlap-extension method^60^, which joins two 400–600 bp PCR fragments corresponding to genomic sequences flanking the gene(s) of interest. The deletion cassettes were then cloned into a suicide plasmid (pDM4)^61^ by using standard cloning procedures followed by DNA sequencing confirmation (Table S2). The resulting constructs were individually electroporated into *E. coli* S17-1 λpir, after which the constructs were introduced by conjugation into MccPDI-producing *E. coli*-25. Mutant strains were selected on LB plates containing Cm and Tet followed by a 10% sucrose selection process. Gene deletion was confirmed by PCR using primers located just outside of the deleted sequence (Table S2).

Plasmids for complementation (pMMB207 and pCR2.1, Table S3) and overexpression (pPAL7, Table S3) were constructed by using standard cloning procedures and all inserts were fully sequenced to confirm construct assembly. For site-directed mutagenesis, primers (Table S2) were designed by using NEBaseChanger (http://nebasechanger.neb.com/) and were then used to generate point mutation plasmids (Table S3) with a Q5 Site-Directed Mutagenesis Kit (New England Biolabs) following the manufacturer’s protocol. These constructs were introduced into their target strains by electroporation.

### Competition assays

Bacterial strains were grown individually overnight in LB media with appropriate antibiotics. Equal volumes of each competing strain were inoculated at 1:200 into either fresh LB medium with different salt concentrations or M9 medium. The cultures were mixed and incubated at 37 °C for 12 h. When necessary, IPTG (100 μM unless specified otherwise) and antibiotics (chloramphenicol or ampicillin) were added during the competition. Monocultures of each competing strain were also prepared as controls by inoculation into the appropriate media at the same dilution. To estimate the number of colony forming units (CFUs) for each strain following competition, a 6 × 6 drop-plate method was employed^62^ with triplicate counts for each competition experiment (technical replicates were averaged before analysis).

### RNA isolation and qPCR

Expression was quantified for *mcpM, mcpI, mcpA* and *mcpB* at 4, 8, 12 and 24 h. Briefly, a cell pellet was collected by centrifugation from 1.0–1.5 mL broth culture. This was resuspended in RNAwiz reagent (350 μL; Bacteria Ribopure kit; Ambion). Primary organic extraction was carried out as per manufacturer’s instructions. The RNA was treated with RQ1-RNase-free DNase (Promega) for 30 min at 37 °C, followed by a second organic extraction using TRIzol LS (Invitrogen) as per manufacturer’s instructions. The final RNA was quantified using a NanoDrop™ 2000 Spectrophotometer (Thermo Scientific). All RNA extractions were confirmed as “DNA free” by subjecting them to a qPCR reaction with primers for *rpoD* (without cDNA synthesis). Any samples for which a Ct value of <38 cycles was generated were treated a second time with DNase and were re-extracted as described above. RT reactions were performed utilizing iScript Supermix (BioRad) as per manufacturer’s instructions with 500 ng of RNA in a total volume of 20 μL. The resultant cDNA was diluted 1:10 with the addition of 180 μL of ultra-pure water. Diluted cDNA (5 μL) was used as template in each qPCR reaction.

qPCR reactions included 10 μL of SsoAdvance SYBR Mastermix (2X) (BioRad),. 5 μL of cDNA template and 200 nM of each primer in a final volume of 20 μL. All primer pairs (Table S2) were run using the same cycling parameters: initial denaturation at 95 °C for 2 min, followed by 40 cycles of 55 °C for 1 min and 95 °C for 15 s. Fluorescent signal was recorded during the annealing/extension step (55 °C). A melt-curve analysis was performed on all reactions starting at 75 °C and increasing 0.5 °C/cycle, with a pause and fluorescence detection at each temperature for 5 s. All assays were run in triplicate and each condition was run in biological duplicate. *rpoD* served as the housekeeping gene for normalization purposes.

### Electrophoretic mobility shift assays (EMSA)

DNA fragments 1–8 (see results) were prepared by PCR and were then purified by using a QIAquick PCR purification kit (Qiagen). Fragments 9–10 were obtained by annealing oligonucleotides in annealing buffer (10 mM Tris pH 8.0, 50 mM NaCl, 1 mM EDTA). Briefly, equal volumes of complementary oligonucleotides (at equimolar concentration) were mixed in a 1.5 ml microfuge tube and placed in a heat block at 95 °C for 5 min. The heat block, along with the samples, was removed from the apparatus and allowed to cool for 1 h to room temperature. The resulting double-stranded DNA was separated on a 2.0% agarose gel and purified using the QIAquick gel extraction kit (Qiagen). All fragments were quantified using a NanoDrop™ 2000 Spectrophotometer prior to performing EMSA experiments. The OmpR and XRE (control) proteins were expressed and purified using the Profinity eXact System as described in detail elsewhere (Fig. S2). Concentration of purified proteins was estimated using a micro-BCA protein assay kit (Thermo Scientific). The purified proteins were mixed with the DNA fragments at different concentrations in 20 μL of binding buffer [10 mM Tris (pH 7.5), 100 mM KCl, 10 mM MgCl_2_, 1 mM DTT, 5% glycerol]. For dephosphorylation experiments, OmpR was dephosphorylated using calf-intestinal alkaline phosphatase (NEB) as per manufacturer’s instructions. Binding reactions were incubated at room temperature for 30 min before adding 5 μL of 5X loading buffer. The samples were electrophoresed on 5% native TBE gels (BioRad) for 45 min at 100 V followed by staining with ethidium bromide.

### Western blot analysis

Protein samples from bacterial pellets and cell fractions were denatured in boiling water for 5 min in tricine sample buffer (BioRad). SDS-PAGE was used to separate proteins with either Any kD Tris-glycine precast gels or a 16.5% Tris-Tricine precast gels (BioRad) prior to western blotting. The Tris-Tricine gels were used to improve resolution for McpM. A Trans-Blot turbo transfer starter system (BioRad) was used to transfer proteins to a low-fluorescence polyvinylidene fluoride (LF-PVDF) membrane. Primary antibodies anti-His-tag (1:2500, Novagen), anti-DnaK (1:5000, Abcam) were used with secondary goat anti-mouse antibody (1:5000, DyLight 488 conjugate) to visualize proteins on western blots. A ChemiDoc MP Imaging System (BioRad) was used to detect fluorescent signal.

### Supernatant protein precipitation

Bacterial strains were grown 10 h at 37 °C (200 rpm) in M9 broth (50 ml) supplemented with appropriate antibiotics. Supernatants were filtered through 0.45-μm PVDF syringe filters, and the proteins in the supernatant were precipitated by adding 20% (vol/vol) trichloroacetic acid (TCA) followed by incubation on ice for 1 h. Precipitated protein was pelleted by centrifugation (12,000 × *g* for 1 h), washed with acetone for 15 min, dried, and suspended in Tricine sample buffer.

### Statistical analysis

All qPCR results were processed using the ∆-∆ Ct method^63^ with the resultant fold change/biological replicate analyzed using an ANOVA with a Bonferroni multiple comparison post-hoc test (NCSS 2007; LLC. Kaysville, UT). Other comparisons were made by using ANOVA with a Dunnett’s one-way multiple comparisons post-hoc test (SigmaPlot version 12.5; Systat Software, Inc., San Jose, CA).

## Additional Information

**How to cite this article**: Zhao, Z. *et al*. Microcin PDI regulation and proteolytic cleavage are unique among known microcins. *Sci. Rep.* **7**, 42529; doi: 10.1038/srep42529 (2017).

**Publisher's note:** Springer Nature remains neutral with regard to jurisdictional claims in published maps and institutional affiliations.

## Supplementary Material

Supplementary Information
