# Supplementary material for: Microcin PDI regulation and proteolytic cleavage are unique among known microcins
Source: Sci Rep. 2017 Feb 16;7:42529. doi: 10.1038/srep42529 (PMC5311971; doi:10.1038/srep42529)
Supplement: Supplementary Information [file srep42529-s1.pdf]

Supplemental Figures

Figure. S1. (A) Transcription analysis of the mccPDI genes (*mcpI*, *mcpA* and *mcpB*) using qPCR after culture in LB or M9 media. All fold changes are expressed relative to gene expression in LB at 24 hr. $^{a}P < 0.05$ when compared to all LB time points and for the 4 and 12 h measurements for culture in M9 media. $^{b}P < 0.05$ when compared to all other time points (LB or M9). Statistical significance was assessed using a single-factor ANOVA followed by a Bonferroni multiple comparison test. Error bars = SEM for three independent replicates. (B) Genetic organization of the PDI gene cluster which shows the length of non-coding region between the mccPDI genes.

(A)

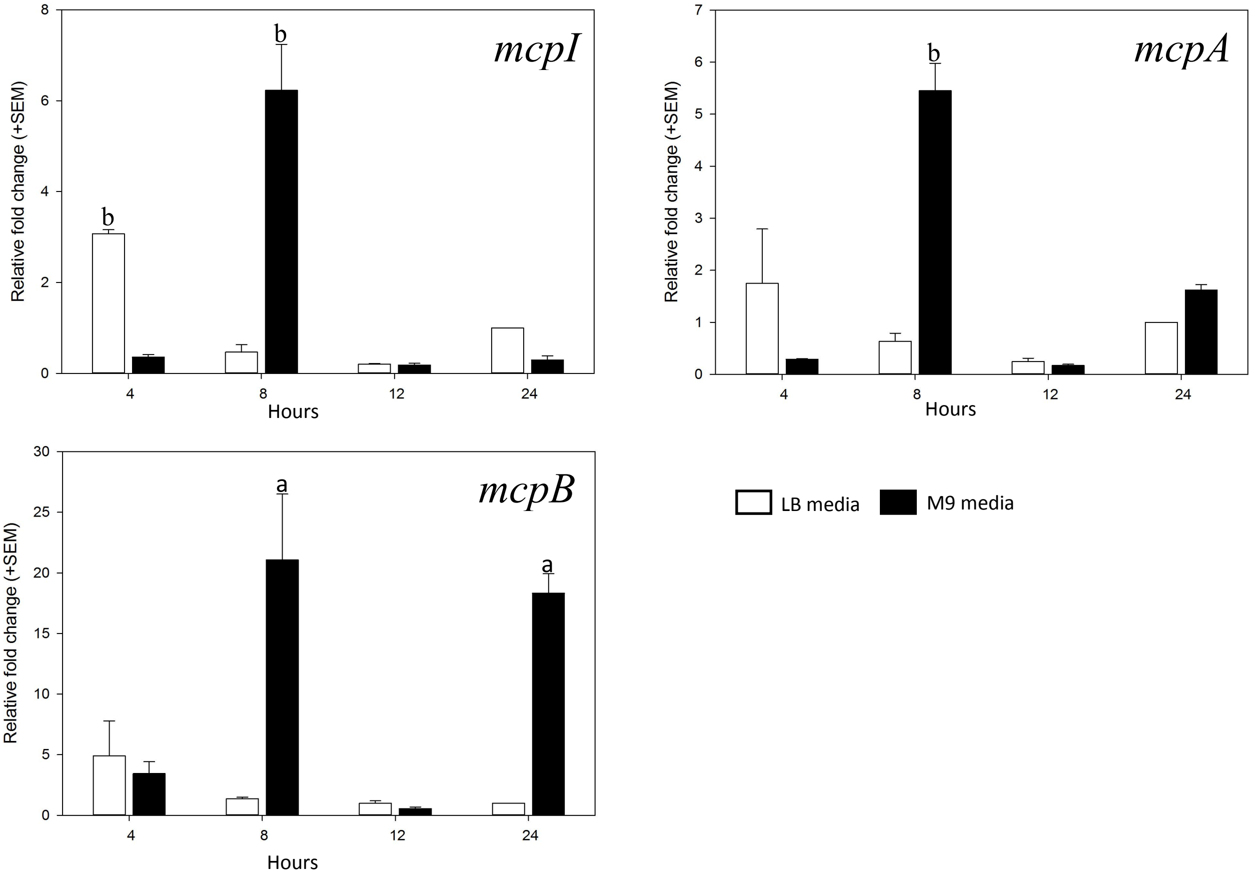

(B)

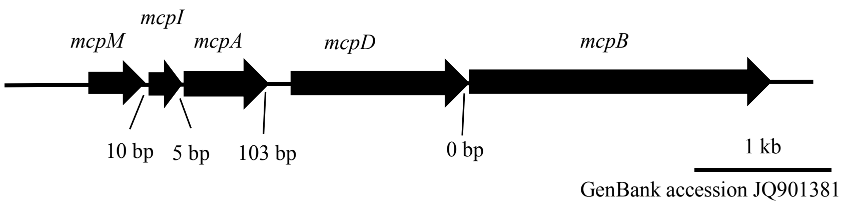

Figure. S2. Expression and purification of OmpR and Xenobiotic Responsive Element (XRE) proteins. Briefly, the OmpR and XRE gene were individually cloned into the pPAL7 vector to construct the recombinant plasmids pPAL-OmpR and pPAL-XRE. These were then transformed into *E. coli* BL21 (DE3). These strains were induced in LB for 5 h with 0.2 mM IPTG at room temperature. Induced cells (20 ml cultures) were harvested by centrifugation at 3200×*g* for 10 min. The pellets were lysed by adding 600 µl of BugBuster master mix (Novagen) for 20 min at room temperature. The soluble fraction was separated by centrifugation at 18,000×*g* for 15 min, and was then subjected to affinity chromatography using the Profinity eXact purification system following the manufacturer's instructions (Bio-Rad). Samples were assessed using electrophoresis with AnykD TGX precast gels (Bio-Rad). Profinity eXact affinity-tagged proteins are indicated by green arrow while red arrows show the tag-free protein. Lane 1, Precision Plus Protein standards; lane 2, crude lysate from *E. coli* carrying pPAL-OmpR; lane 3, soluble fraction from *E. coli* carrying pPAL-OmpR; lane 4, purified tag-free OmpR; lane 5, crude lysate from *E. coli* carrying pPAL-XRE; lane 6, soluble fraction from *E. coli* carrying pPAL-XRE; lane 7, purified tag-free XRE.

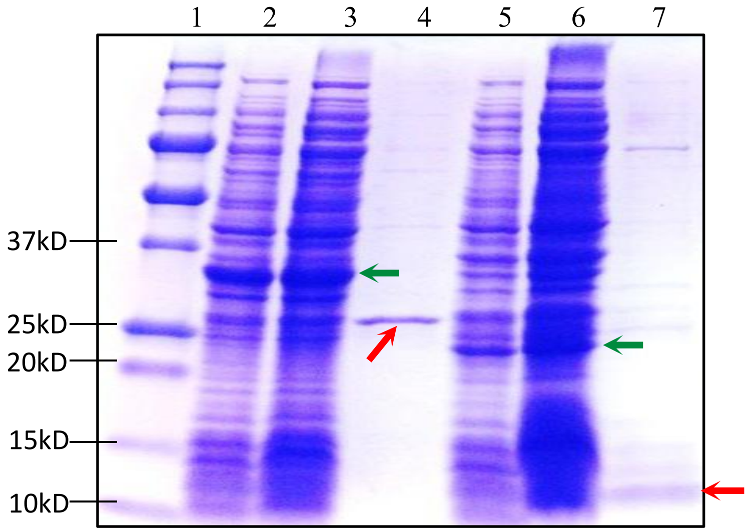

Figure. S3. Electrophoretic Gel Mobility shift assay (EMSA). (A) A 270 bp unrelated control DNA fragment (*atpE*), a 201 bp DNA fragment ($P_{mic\text{-}233/\text{-}433}$) located at position from -233 bp to -433 bp relative to the start codon of *mcpM*, and a 143 bp DNA fragment ($P_{micD\text{-}20/\text{-}163}$) located at position from -20 bp to -163 bp relative to the start codon of *mcpD*, were subjected to EMSA using OmpR expressed and purified from LB both (Fig. S2). No shift was evident for these DNA fragments indicating no evidence that OmpR binds to these regions. (B) A representative gel showing binding of OmpR with $P_{mic\text{-}10/\text{-}210}$ and loss of binding when OmpR was dephosphorylated by using calf intestinal alkaline phosphatase (CIP). 300 ng OmpR was used in this assay.

(A)

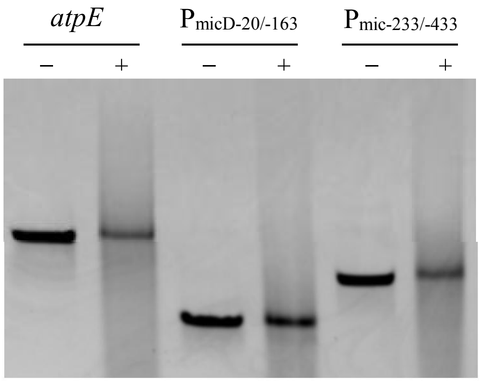

(B)

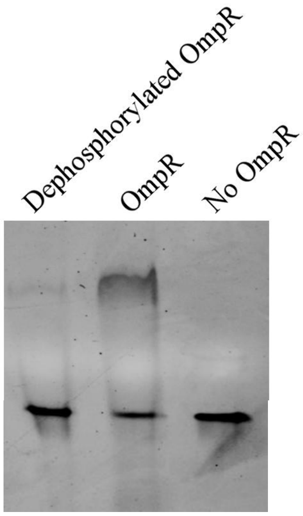

Figure. S4. (A) Amino acid sequence alignment of class II microcin precursors. McsS, microcin S (YP_006954535); ColV, colicin V (CAA40746); MccL microcin L (AAP03989); Mcc24, microcin 24 (AAA88772); MccE492, microcin E492 (AAD04332); MccH47, MchB protein (CAB54534). The red arrows indicate the cleavage sites corresponding to McpM that we identified in this study. The inverted triangles indicate the position of the four cysteines of McpM. Sequence alignments were generated using ClustalX 1.83. Parenthetical numbers on the right indicate the amino acid position relative to the N terminus of each sequence. The black shaded regions indicate completely conserved residues, while the grey shaded regions are partially conserved residues with greater than 60% identity. (B) Western blot analysis of McpM after replacement of specific residues in double-knockout strain E25Δ*mcpM*Δ*mcpA*. (C) Competition assays between the different residue-specific mutants and BW25113 (vector ctrl). Competition was performed in M9 medium with chloramphenicol (34 µg/ml) and 0.5 mM IPTG for 12 h. Results are expressed as the difference in CFUs of the sensitive strain grown in co-culture and monoculture.

(A)

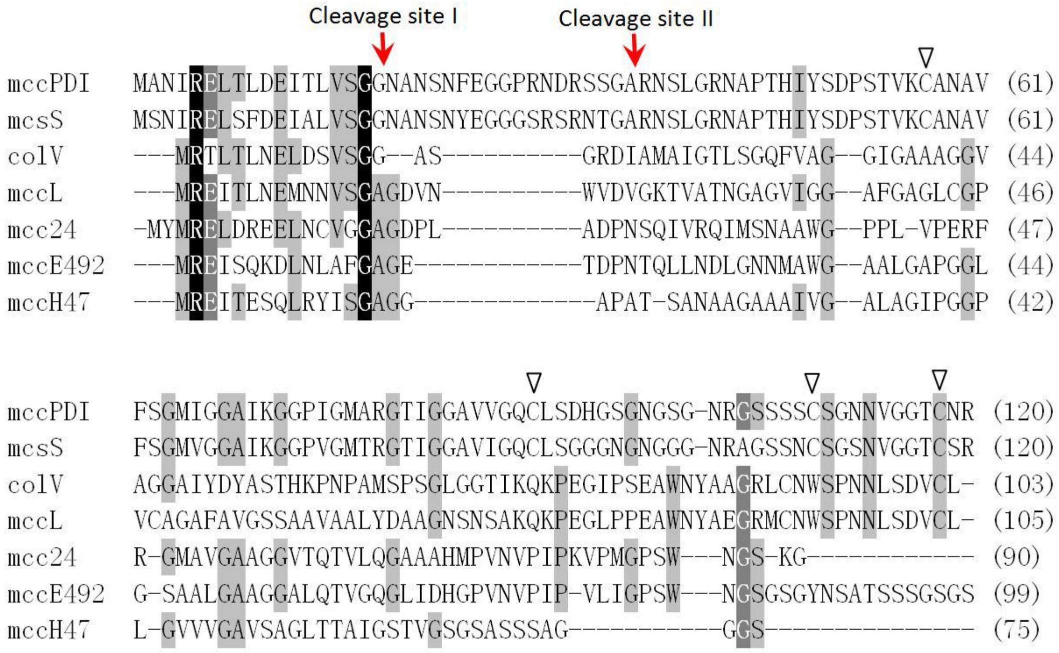

(B)

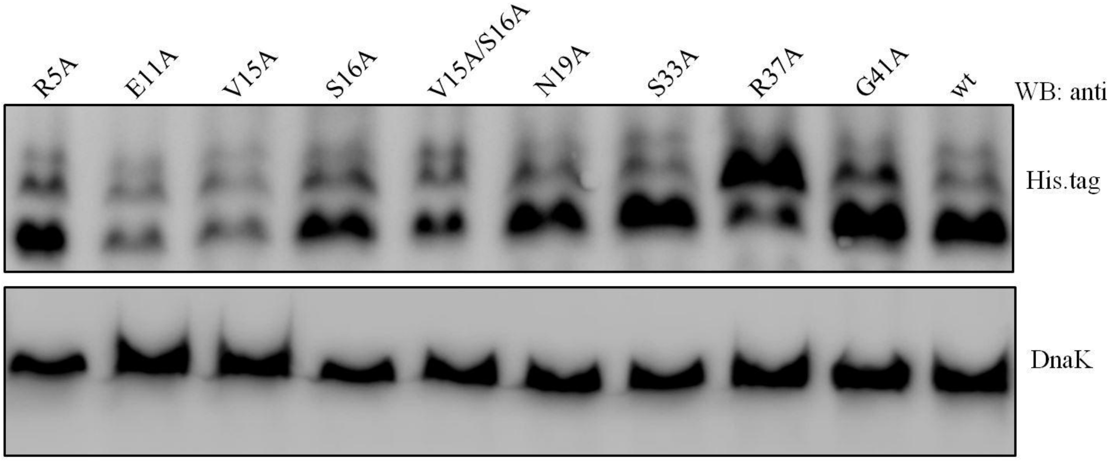
R5A
E11A
V15A
S16A
V15A/S16A
N19A
S33A
R37A
G41A
wt
WB: anti
His.tag
DnaK

(C)

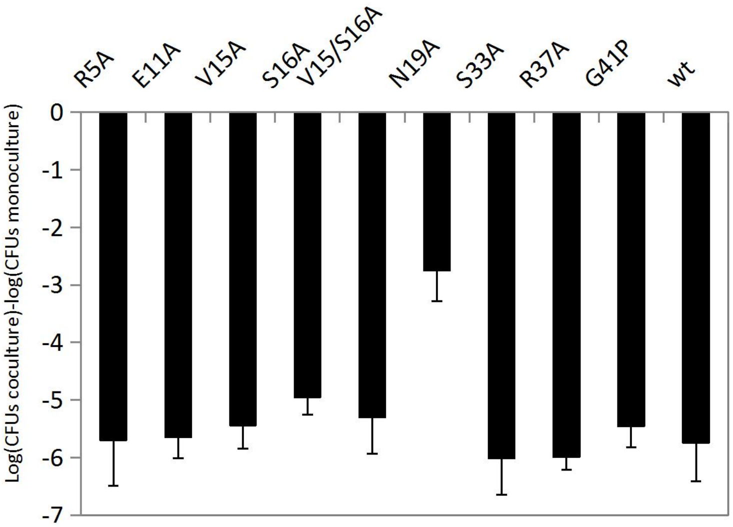
R5A
E11A
V15A
S16A
V15/S16A
N19A
S33A
R37A
G41P
wt
Log(CFUs coculture)-log(CFUs monoculture)
0
-1
-2
-3
-4
-5
-6
-7

Figure. S5. Western blot of McpM from four different constructs (Δ1-18, Δ1-36, Δ19-36, and colV1-15/Δ19-36) in strain E25Δ*mcpM* Δ*mcpA*. Wild-type E25 (McpM producing; wt) was used as positive control. Three bands, indicated by black arrows adjacent to the first lane, correspond to the putative full length McpM (top) followed by two cleaved forms of the protein.

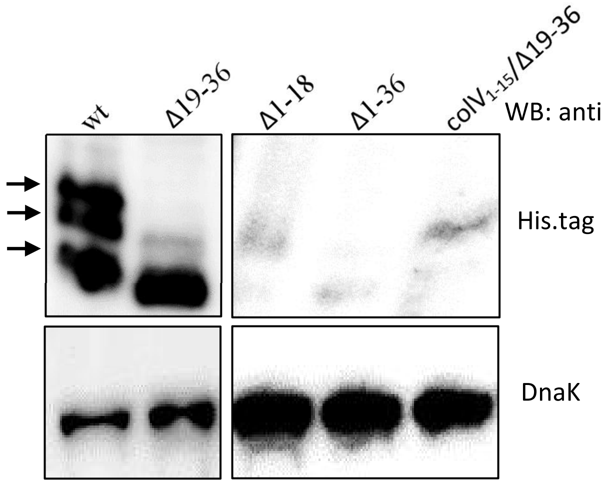

Figure. S6. Disulfide bond formation system in *E. coli* is not involved in mccPDI activity in producing strains. CFUs of *E. coli* BW25113 following competition with wild-type E25 and different knockouts strains in the E25 background (single and double). Results are expressed as the difference in CFUs of the sensitive strain grown in co-culture and monoculture. Experiments were done in duplicate with error bars representing the standard error of the mean (SEM). *, statistically significant ANOVA (*P* <0.01 with Dunnett's upper one-sided multiple-comparison test with control).

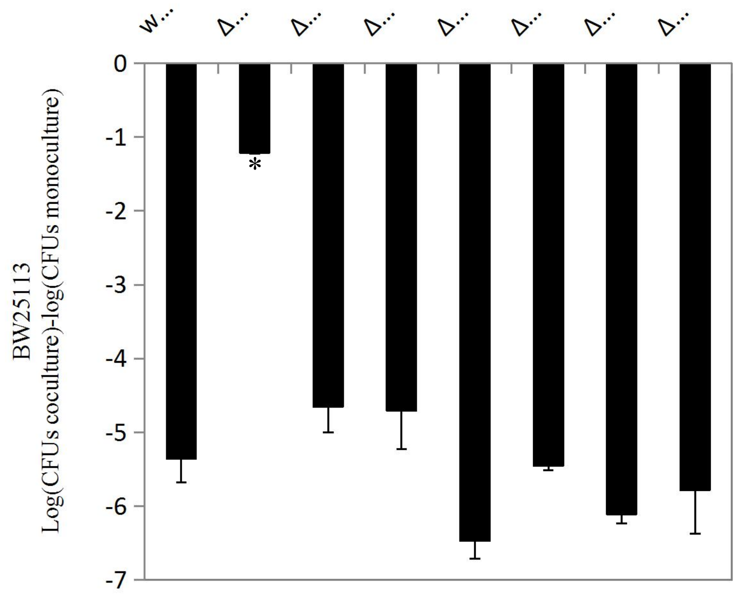

Supplemental Table S1. *E.coli* strains used in this study

Strains name	Designation	Relevant genotype/phenotype	Reference
E. coli-25	E25	Wild type; SSuT^r PDI$^+$	Sawant et al., 2011
E25Δ*envZ*	Δ*envZ*	SSuT^r PDI$^-$, *envZ* knockout	This study
E25Δ*ompR*	Δ*ompR*	SSuT^r PDI$^-$, *ompR* knockout	This study
E25Δ*envZ*(p207::*envZ*)	Δ*envZ*(*envZ*)	*envZ* knockout complemented with *envZ*	This study
E25Δ*envZ*(p207::*envZ* H243A)	Δ*ompR*(*envZ* H243A)	*envZ* knockout complemented with mutant *envZ*(H243A)	This study
E25Δ*ompR*(p207::*ompR*)	Δ*ompR*(*ompR*)	*envZ* knockout complemented with *ompR*	This study
E25Δ*mcpM*	Δ*mcpM*	SSuT^r PDI$^-$, *mcpM* knockout	This study
E25Δ*mcpM*Δ*mcpA*	Δ*mcpM&A*	SSuT^r PDI$^-$, *mcpM and mcpA* double-knockout	This study
E25Δ*mcpB*	Δ*mcpB*	SSuT^r PDI$^-$, *mcpB* knockout	This study
E25Δ*mcpD*	Δ*mcpD*	SSuT^r PDI$^-$, *mcpD* knockout	This study
E25Δ*mcpM*Δ*mcpB*	Δ*mcpM&B*	SSuT^r PDI$^-$, *mcpM* and *mcpB* double-knockout	This study
E25Δ*mcpM*Δ*mcpD*	Δ*mcpM&D*	SSuT^r PDI$^-$, *mcpM* and *mcpD* double-knockout	This study
E25Δ*mcpM*(p207::*mcpM*)	Δ*mcpM*(*mcpM*)	*mcpM* knockout complemented with *mcpM* driven by tac promoter	This study
E25Δ*mcpM*(pCR2.1:: $P_{mic-1/-210}$+*mcpM*)	Δ*mcpM*(P_{mic}+*mcpM*)	*mcpM* knockout complemented with *mcpM* driven by the endogenous promoter	This study
E25Δ*mcpB*(p207::*mcpB*)	ΔmcpB(*mcpB*)	*mcpB* knockout complemented with *mcpB* driven by tac promoter	This study
E25Δ*mcpD*(p207::*mcpD*)	Δ*mcpD*(*mcpD*)	*mcpM* knockout complemented with *mcpD* driven by tac promoter	This study
E25Δ*mcpM*Δ*mcpB*(p207::*mcpM*)	Δ*mcpM&B*(*mcpM*)	*mcpM* and *mcpB* double-knockout complemented with *mcpM* driven by tac promoter	This study
E25Δ*mcpM*Δ*mcpD*(p207::*mcpM*)	Δ*mcpM&D*(*mcpM*)	*mcpM* and *mcpD* double-knockout complemented with *mcpM* driven by tac promoter	This study
E25Δ*mcpM*Δ*mcpA* (p207::*mcpM* R5A)	Δ*mcpM&A*(*mcpM* R5A)	*mcpM* and *mcpA* double-knockout complemented with point mutant *mcpM* (R5A)	This study
E25Δ*mcpM*Δ*mcpA* (p207::*mcpM* E11A)	Δ*mcpM&A*(*mcpM* E11A)	*mcpM* and *mcpA* double-knockout complemented with point mutant *mcpM* (E11A)	This study
E25Δ*mcpM*Δ*mcpA* (p207::*mcpM* V15A)	Δ*mcpM&A*(*mcpM* V15A)	*mcpM* and *mcpA* double-knockout complemented with point mutant *mcpM* (V15A)	This study
E25Δ*mcpM*Δ*mcpA* (p207::*mcpM* S16A)	Δ*mcpM&A*(*mcpM* S16A)	*mcpM* and *mcpA* double-knockout complemented with point mutant *mcpM* (S16A)	This study
E25Δ*mcpM*Δ*mcpA* (p207::*mcpM* V15A/S16A)	Δ*mcpM&A*(*mcpM* V15A/S16A)	*mcpM* and *mcpA* double-knockout complemented with point mutant *mcpM* (V15A/S16A)	This study
E25Δ*mcpM*Δ*mcpA* (p207::*mcpM* G17A)	Δ*mcpM&A*(*mcpM* G17A)	*mcpM* and *mcpA* double-knockout complemented with point mutant *mcpM* (G17A)	This study
E25Δ*mcpM*Δ*mcpA* (p207::*mcpM* G18A)	Δ*mcpM&A*(*mcpM* G18A)	*mcpM* and *mcpA* double-knockout complemented with point mutant *mcpM* (G18A)	This study
E25Δ*mcpM*Δ*mcpA* (p207::*mcpM* G17A/G18A)	Δ*mcpM&A*(*mcpM* G17A/G18A)	*mcpM* and *mcpA* double-knockout complemented with point mutant *mcpM* (G17/18A)	This study
E25Δ*mcpM*Δ*mcpA* (p207::*mcpM* G17P)	Δ*mcpM&A*(*mcpM* G17P)	*mcpM* and *mcpA* double-knockout complemented with point mutant *mcpM* (G17P)	This study
E25Δ*mcpM*Δ*mcpA* (p207::*mcpM* G18P)	Δ*mcpM&A*(*mcpM* G18P)	*mcpM* and *mcpA* double-knockout complemented with point mutant *mcpM* (G18P)	This study
E25Δ*mcpM*Δ*mcpA* (p207::*mcpM* N19A)	Δ*mcpM&A*(*mcpM* N19A)	*mcpM* and *mcpA* double-knockout complemented with point mutant *mcpM* (N19A)	This study
E25Δ*mcpM*Δ*mcpA* (p207::*mcpM* G26P)	Δ*mcpM&A*(*mcpM* G26P)	*mcpM* and *mcpA* double-knockout complemented with point mutant *mcpM* (G26P)	This study
E25Δ*mcpM*Δ*mcpA* (p207::*mcpM* G27P)	Δ*mcpM&A*(*mcpM* G27P)	*mcpM* and *mcpA* double-knockout complemented with point mutant *mcpM* (G27P)	This study
E25Δ*mcpM*Δ*mcpA* (p207::*mcpM* S33A)	Δ*mcpM&A*(*mcpM* S33A)	*mcpM* and *mcpA* double-knockout complemented with point mutant *mcpM* (S33A)	This study
E25Δ*mcpM*Δ*mcpA* (p207::*mcpM* G35P)	Δ*mcpM&A*(*mcpM* G35P)	*mcpM* and *mcpA* double-knockout complemented with point mutant *mcpM* (G35P)	This study
E25Δ*mcpM*Δ*mcpA* (p207::*mcpM* A36D)	Δ*mcpM&A*(*mcpM* A36D)	*mcpM* and *mcpA* double-knockout complemented with point mutant *mcpM* (A36D)	This study
E25Δ*mcpM*Δ*mcpA* (p207::*mcpM* R37A)	Δ*mcpM&A*(*mcpM* R37A)	*mcpM* and *mcpA* double-knockout complemented with point mutant *mcpM* (R37A)	This study
E25Δ*mcpM*Δ*mcpA* (p207::*mcpM* G41P)	Δ*mcpM&A*(*mcpM* G41P)	*mcpM* and *mcpA* double-knockout complemented with point mutant *mcpM* (G41P)	This study
E25Δ*mcpM*Δ*mcpA* (p207::*mcpM* C57A)	Δ*mcpM&A*(*mcpM* C57A)	*mcpM* and *mcpA* double-knockout complemented with point mutant *mcpM* (C57A)	This study
E25Δ*mcpM*Δ*mcpA* (p207::*mcpM* C90A)	Δ*mcpM&A*(*mcpM* C90A)	*mcpM* and *mcpA* double-knockout complemented with point mutant *mcpM* (C90A)	This study
E25Δ*mcpM*Δ*mcpA* (p207::*mcpM* C109A)	Δ*mcpM&A*(*mcpM* C109A)	*mcpM* and *mcpA* double-knockout complemented with point mutant *mcpM* (C109A)	This study
E25Δ*mcpM*Δ*mcpA* (p207::*mcpM* C118A)	Δ*mcpM&A*(*mcpM* C118A)	*mcpM* and *mcpA* double-knockout complemented with point mutant *mcpM* (C118A)	This study
E25Δ*mcpM*Δ*mcpA* (p207::*mcpM* Δ1-18)	Δ*mcpM&A*(*mcpM* Δ1-18)	*mcpM* and *mcpA* double-knockout complemented with mutant *mcpM* (1-18 deletion)	This study
E25Δ*mcpM*Δ*mcpA* (p207::*mcpM* Δ1-36)	Δ*mcpM&A*(*mcpM* Δ1-36)	*mcpM* and *mcpA* double-knockout complemented with mutant *mcpM* (1-36 deletion)	This study

E25Δ*mcpM*Δ*mcpA* (p207::*mcpM* Δ19-36)	Δ*mcpM&A*(*mcpM* Δ19-36)	*mcpM* and *mcpA* double-knockout complemented with mutant *mcpM* (19-36 deletion)	This study
E25Δ*mcpM*Δ*mcpA* (p207::$ColV_{1\text{-}15}$+mcpMΔ1-18)	Δ*mcpM&A*(*ColV*1-15/Δ1-18)	*mcpM* and *mcpA* double-knockout complemented with the recombinant mcpM (replacement of the first leader peptide sequence1-18 by colicin V leader sequence)	This study
E25Δ*dsbA*	Δ*dsbA*	SSuT^r PDI$^+$, *dsbA* knockout	This study
E25Δ*dsbB*	Δ*dsbB*	SSuT^r PDI$^+$, *dsbB* knockout	This study
E25Δ*dsbA*Δ*dsbB*	Δ*dsbA* &*B*	SSuT^r PDI$^+$, *dsbA* and *dsbB* double-knockout	This study
E25Δ*dsbD*	Δ*dsbD*	SSuT^r PDI$^+$, *dsbD* knockout	This study
E25Δ*dsbA*Δ*dsbD*	Δ*dsbA* &*D*	SSuT^r PDI$^+$, *dsbA* and *dsbD* double-knockout	This study
E25Δ*dsbA*Δ*dsbB*Δ*dsbD*	Δ*dsbA* &*B* &D	SSuT^r PDI$^+$, *dsbA, dsbB* and *dsbD* triple-knockout	This study
E25Δ*traM::*Cm	Δ*traM::*Cm	SSuT^r PDI$^+$, *traM* knockout with a replacement of chloramphenicol cassette	Eberhart et al., 2014
E25Δ*traM::*Kan	Δ*traM::*Kan	SSuT^r PDI$^+$, *traM* knockout with a replacement of kanamycin cassette	Eberhart et al., 2012
***E. coli* BW25113**	BW	Nal^r, Keio collection wild-type K-12 strain	Baba et al., 2006
BW25113 (pMMB207 only)	BW(vector ctrl)	Nal^r Cm^r, BW25113 with empty pMMB207 vector	Zhao et al, 2015
BW25113 (p207::*mcpM*)	BW(*mcpM*)	Nal^r Cm^r, BW25113 with recombinant plasmid p207::*mcpM*	This study
***E. coli* 186**	186	Wild type; Nal^r PDI$^-$, susceptible strain to microcin PDI.	Eberhart et al.,2012
**186(** p207::*mcpM*)	186(*mcpM*)	Nal^r Cm^r, 186 with recombinant plasmid p207::*mcpM*	This study
E. coli* S17-1 λ *pir	S17	*thi pro hsdR hsdM$^+$recA* RP4-2-Tc::Mu-Km::Tn7 *λpir* lysogen	Simon et al.(1983)
S17:pDM4-Δ*envZ*(A1+A2)	S17:pDM4-Δ*envZ*	S17 strain carrying the plasmid pDM4-Δ*envZ*	This study
S17:pDM4-Δ*ompR*(A1+A2)	S17:pDM4-Δ*ompR*	S17 strain carrying the plasmid pDM4-Δ*ompR*	This study
S17:pDM4-Δ*mcpM*(A1+A2)	S17:pDM4-Δ*mcpM*	S17 strain carrying the plasmid pDM4-Δ*mcpM*	This study
S17:pDM4-Δ*mcpB*(A1+A2)	S17:pDM4-Δ*mcpB*	S17 strain carrying the plasmid pDM4-Δ*mcpB*	This study
S17:pDM4-Δ*mcpD*(A1+A2)	S17:pDM4-Δ*mcpD*	S17 strain carrying the plasmid pDM4-Δ*mcpD*	This study
S17:pDM4-Δ*dsbA*(A1+A2)	S17:pDM4-Δ*dsbA*	S17 strain carrying the plasmid pDM4-Δ*dsbA*	This study
S17:pDM4-Δ*dsbB*(A1+A2)	S17:pDM4-Δ*dsbB*	S17 strain carrying the plasmid pDM4-Δ*dsbB*	This study
S17:pDM4-Δ*dsbD*(A1+A2)	S17:pDM4-Δ*dsbD*	S17 strain carrying the plasmid pDM4-Δ*dsbD*	This study
***E. coli* BL21(DE3)**	BL21	F$^-$ *ompT* $hsdS_B(r_B^-, m_B^-)$ *gal dcm* (*DE3*)	Invitrogen
BL21(DE3):pPAL7-*ompR*	BL21:pPAL7-*ompR*	BL21 (DE3) carrying the plasmid pPAL7-*ompR*	This study
BL21(DE3):pPAL7-*ompR* D55A	BL21:pPAL7-*ompR* D55A	BL21 (DE3) carrying the plasmid pPAL7-*ompR* D55A	This study
BL21(DE3):pPAL7-*XRE*	BL21:pPAL7-*XRE*	BL21 (DE3) carrying the plasmid pPAL7-*XRE*	This study

Supplemental Table S2. PCR primers used in this study

Primer name	Sequence(5’_3’)*	Usage
mcpM_A1_BglII<br>mcpM_A1_R<br>mcpM_A2_F<br>mcpM_A2_SalI	GGAAGATCTATGGCAATGCTGGAAGAG<br>CATTATTACCATCATATTTCCCCTATCGGT<br>GAAATATGATGGTAATAATGTTGGCGGAAC<br>ACGCGTCGACATGAGAGCAATCACAGCAA	construct suicide plasmid pDM4_Δ*mcpM*(A1+A2) for mcpM knockout
mcpM_int_F:<br>mcpM_int_R:	AGATGAGATAACGCTTGTCA<br>ACTTCCTCTGTTACCACTTC	confirm mcpM knockout
mcpM/I_A1_BglII<br>mcpM/I_A1_R<br>mcpM/I_A2_F<br>mcpM/I_A2_SalI	GGAAGATCTATGGCAATGCTGGAAGAG<br>AATCTTGCCGATCATATTTCCCCTATCGGT<br>GAAATATGATCGGCAAGATTCATGGACTA<br>ACGCGTCGACCTTCATAATACGGAACTGTCAG	construct suicide plasmid pDM4_Δ*mcpM/I*(A1+A2) for mcpM & mcpI knockout
mcpI_int_F<br>mcpI_int_R	TATGTGGTTTGTTACTGGGAT<br>CGCGGAGATTGTTCTTATTT	confirm mcpI knockout
mcpA_A1_SacI<br>mcpA_A1_R<br>mcpA_A2_F<br>mcpA_A2_SalI	GGAGAGCTCGATGAGATAACGCTTGTCAG<br>AGTTTTATACAATGCTATTTCGCGGAGAT<br>AAATAGCATTGTATAAAACTTTAACATCACACA<br>ACGCGTCGACGGCTTCCTGTTGTTGACTA	construct suicide plasmid pDM4_Δ*mcpA*(A1+A2) for mcpA knockout
mcpA_int_F<br>mcpA_int_R	CGGCATTCACCATACAATA<br>CATAATACGGAACTGTCAGG	confirm mcpA knockout
mcpD_A1_SacI<br>mcpD_A1_R<br>mcpD_A2_F<br>mcpD_A2_SalI	GGAGAGCTCTTGCTGTGATTGCTCTCAT<br>ACAGATTTCCAAGTAACCTTTCCGTCAACA<br>AAGGTTACTTGGAAATCTGTAATGGAATCA<br>ACGCGTCGACTCACTGGCTGGAGTTAATTC	construct suicide plasmid pDM4_Δ*mcpD*(A1+A2) for mcpA knockout
mcpD_int_F<br>mcpD_int_R	TCAACAACAGGAAGCCATA<br>AAGGAGCCAGAGTCGTAT	confirm mcpD knockout
mcpB_A1_SacI<br>mcpB_A1_R<br>mcpB_A2_F<br>mcpB_A2_SalI	GGAGAGCTCCAATGGACACAGCCAAAGA<br>GCGGATGCTATACAGATTTCCTTTCATGCTCC<br>GAAATCTGTATAGCATCCGCAGACAGAGTT<br>ACGCGTCGACACCCGTTGATTTATGTGAGA	construct suicide plasmid pDM4_Δ*mcpB*(A1+A2) for mcpA knockout
mcpB_int_F<br>mcpB_int_R	CCATTTCGTCGTTCTCCATA<br>TTGCCACATCCTGATTTACC	confirm mcpB knockout
mcpM_p207_EcoRI<br>mcpM_p207_SalI	CCGGAATTCATGGCAAATATAAGAGA<br>ACGCGTCGACTTAATGGTGATGGTGATGATGTCGGTTACATGTTCCGCCA	construct recombinant plasmid p207::mcpM for mcpM expression under tac promoter control
mcpD_p207_SacI<br>mcpD_p207_SalI	GGAGAGCTCATGAATATATTCAGAAGTGA<br>ACGCGTCGACTTAATGGTGATGGTGATGATGCAGATTTCCTTTCATGCTCCA	construct recombinant plasmid p207::mcpD for mcpD expression under tac promoter control
mcpB_p207_EcoRI<br>mcpB_p207_SalI	GGAGAGCTCATGGAATCAATAAACTGGA<br>GCAGGTCGACTCAATGGTGATGGTGATGATGCCTCCTGTTGGGGTGATTA	construct recombinant plasmid p207::mcpB for mcpB expression under tac promoter control

Δ1-18_p207_EcoRI<br>Δ1-18_p207_SalI	CCGGAATTCATGAACGCAAACAGCAACTTTGA<br>ACGCGTCGACTTAATGGTGATGGTGATGATGTCGGTTACATGTTCCGCCA	construct recombinant plasmid<br>p207:: *mcpM* Δ1-18for mutant mcpM expression (1-18 deletion) under tac promoter control
Δ1-36_p207_EcoRI<br>Δ1-36_p207_SalI	CCGGAATTCATGCGTAACTCACTGGGTCGAA<br>ACGCGTCGACTTAATGGTGATGGTGATGATGTCGGTTACATGTTCCGCCA	construct recombinant plasmid<br>p207:: *mcpM* Δ1-36 for mutant mcpM expression (1-36 deletion) under tac promoter control
Δ19-36_p207_EcoRI<br><br>Δ19-36_p207_SalI	CCGGAATTCATGGCAAATATAAGAGAATTAACTTTAGATGAGATAACGCTTGTCAGCGGAGGAC<br>GTAACTCACTGGGTCGAA<br>ACGCGTCGACTTAATGGTGATGGTGATGATGTCGGTTACATGTTCCGCCA	construct recombinant plasmid<br>p207:: *mcpM* Δ19-36 for mutant mcpM expression (19-36 deletion) under tac promoter control
ColV1-15/Δ1-18_<br>p207_EcoRI<br>Δ1-18_p207_SalI	CCGGAATTCATGAGAACTCTGACTCTAAATGAATTAGATTCTGTTTCTGGTGGTAACGCAAACAG<br>CAACTTTGA<br>ACGCGTCGACTTAATGGTGATGGTGATGATGTCGGTTACATGTTCCGCCA	construct recombinant plasmid<br>p207::$ColV_{1-15}$+$mcpM_{\Delta 1-18}$ for the recombinant mcpM (replacement of mcpM 1-18 by colicin V leader sequence under tac promoter control
R5A_F<br>R5A_R	TGGCAAATATagcaGAATTAACTTTAGATGAG<br>TGAATTCTGTTTCCTGTGTG	generate the replacement of R5 by alanine in the p207:: *mcpM*
E11A_F<br>E11A_R	AACTTTAGATgcgATAACGCTTGTC<br>AATTCTCTTATATTTGCCATGAATTC	generate the replacement of E11 by alanine in the p207:: *mcpM*
V15A_F<br>V15A_R	GATAACGCTTgccAGCGGAGGAA<br>TCATCTAAAGTTAATTCTCTTATATTTGCC	generate the replacement of V15 by alanine in the p207:: *mcpM*
S16A_F<br>S16A_R	TAACGCTTGTcgccGGAGGAAACG<br>TCTCATCTAAAGTTAATTCTCTTATATTTG	generate the replacement of S16 by alanine in the p207:: *mcpM*
V15/S16A_F<br>V15/S16A_R	GATAACGCTTgccgccGGAGGAAACG<br>TCATCTAAAGTTAATTCTCTTATATTTG	generate the replacement of V15 and S16 by alanine in the p207:: *mcpM*
G17A_F<br>G17A_R	GCTTGTCAGCgcaGGAAACGCAA<br>GTTATCTCATCTAAAGTTAATTCTCTTATATTTGC	generate the replacement of G17 by alanine in the p207:: *mcpM*
G18A_F<br>G18A_R	TGTCAGCGGAgcaAACGCAAACA<br>AGCGTTATCTCATCTAAAGTTAATTCTC	generate the replacement of G18 by alanine in the p207:: *mcpM*
G17/18A_F<br>G17/18A_R	GCTTGTCAGCgcagcaAACGCAAACA<br>GTTATCTCATCTAAAGTTAATTCTCTTATATTTG	generate the replacements of G17 and G18 by alanine in the p207:: *mcpM*
G17P_F<br>G17P_R	CGCTTGTCAGcccaGGAAACGCAA<br>TTATCTCATCTAAAGTTAATTCTCTTATATTTG	generate the replacement of G17 by proline in the p207:: *mcpM*
G18P_F<br>G18P_R	TTGTCAGCGGaccaAACGCAAACAG<br>GCGTTATCTCATCTAAAGTTAATTC	generate the replacement of G18 by proline in the p207:: *mcpM*
N19A_F<br>N19A_R	TCAGCGGAGGagccGCAAACAGCA<br>CAAGCGTTATCTCATCTAAAGTTAATTC	generate the replacements of N19 by alanine in the p207:: *mcpM*
G26P_F<br>G26P_R	CAACTTTGAAccaGGCCCCCGTA<br>CTGTTTGCGTTTCCTCCG	generate the replacement of G26 by proline in the p207:: *mcpM*
G27P_F<br>G27P_R	CTTTGAAGGTcccCCCCGTAATG<br>TTGCTGTTTGCGTTTCCTC	generate the replacement of G27 by proline in the p207:: *mcpM*
S33A_F<br>S33A_R	TAATGACCGTgccgctGGGGCTCGTAACTCAC<br>CGGGGGCCACCTTCAAAG	generate the replacement of S33 by alanine in the p207:: *mcpM*
G35P_F<br>G35P_R	ACCGTTCCAGtccgGCTCGTAACTC<br>CATTACGGGGGCCACCTT	generate the replacement of G35 by proline in the p207:: *mcpM*
A36D_F<br>A36D_R	CCGTTCCAGTgaggatCGTAACTCAC<br>TCATTACGGGGGCCACCT	generate the replacement of A36 by aspartic acid in the p207:: *mcpM*

R37A_F<br>R37A_R	CCAGTGGGGCtgctAACTCACTGGGTC<br>AACGGTCATTACGGGGGC	generate the replacement of A36 by alanine in the p207:: *mcpM*
G41P_F<br>G41P_R	GTAACTCACTgcctCGAAACGCACCAACTCATATTTATAG<br>GAGCCCCACTGGAACGGT	generate the replacement of G41 by alanine in the p207:: *mcpM*
C57A_F<br>C57A_R	GCACTGTAAAagccGCTAACGCTG<br>TTGGATCACTATAAATATGAGTTG	generate the replacement of C57 by alanine in the p207:: *mcpM*
C90A_F<br>C90A_R	TTGTTGGTCAagctCTCTCAGATCATGGTAGTGG<br>CGGCTCCACCAATGGTAC	generate the replacement of C90 by alanine in the p207:: *mcpM*
C109A_F<br>C109A_R	GTTCCAGTAGtgctTCAGGTAATAATGTTGGC<br>TTCCTCTGTTACCACTTC	generate the replacement of C109 by alanine in the p207:: *mcpM*
C118A_F<br>C118A_R	TTGGCGGAACagctAACCGACATCATC<br>CATTATTACCTGAACAACTACTG	generate the replacement of C118 by alanine in the p207:: *mcpM*
envZ_A1_SacI<br>envZ_A1_R<br>envZ_A2_F<br>envZ_A2_SalI	GGAGAGCTCGCTGACGACTACATTCCAA<br>ATTTACCCTTGTGACGATGAGCAATAACG<br>TCATCGTCACAAGGGTAAATAAACGGGAGG<br>ACGCGTCGACGGCATTGAAACTATTGTCAGA	construct suicide plasmid pDM4_Δ*envZ* (A1+A2) for envZ knockout
ompR_A1_SacI<br>ompR_A1_R<br>ompR_A2_F<br>ompR_A2_SalI	GGAGAGCTCATTTCACGCAGACGCTTT<br>CACCATGCGGGACCACCAGAATCTTGTAGT<br>TCTGGTGGTCCCGCATGGTGGAAGAAGA<br>ACGCGTCGACCAGACGACAGGCGAACTT	construct suicide plasmid pDM4_Δ*ompR* (A1+A2) for envZ knockout
envZ_207_SacI<br>envZ_207_SalI	GGAGAGCTCATGAGGCGATTGCGCTTCTC<br>ACGCGTCGACTTAATGGTGATGGTGATGATGCCCTTCTTTTGTCGTGCCCTG	construct recombinant plasmid p207::envZ for envZ expression under tac promoter control
envZ_H243A_F<br>envZ_H243A_R	CGGGGGTAAGtgccGACTTGCGCAC<br>CCATCAGCAGCGTGCGGT	generate the replacement of H243 by alanine in the p207:: *envZ*
ompR_207_SacI<br>ompR_207_SalI	GGAGAGCTCATGCAAGAGAACTACAAGA<br>ACGCGTCGACTCAATGGTGATGGTGATGATGTGCTTTAGAGCCGTCCGGT	construct recombinant plasmid p207::*ompR* for ompR expression under tac promoter control
ompR_PAL_SpeI<br>ompR_PAL_EcoRI	TTTGACTAGTATGCAAGAGAACTACAAGA<br>CTGCGAATTCTCAATGGTGATGGTGATGATGTGCTTTAGAGCCGTCCGGT	construct recombinant plasmid pPAL7::*ompR* for ompR expression under tac promoter control
ompR_D55A_F<br>ompR_D55A_R	TTATGGTACTggctTTAATGTTACC<br>GATGGAAAGATTCACGAGTC	generate the replacement of D55 by alanine in the pPAL7::*ompR*
XRE_PAL_SpeI<br>XRE_PAL_EcoRI	TTTGACTAGTATGAAAGATGAATTATTTGCT<br>CTGCGAATTCTTAATGGTGATGGTGATGATGGATTGTTTTCAATTGATTGAT	construct recombinant plasmid pPAL7::*XRE* for XRE expression under tac promoter control
atpA_A1_SacI<br>atpA_A1_R<br>atpA_A2_F<br>atpA_A2_SalI	GGAGAGCTCGTCGTTTATCGCAGTTTGT<br>TCGAGGATGCTAACACCGTCACTTACAGAA<br>GACGGTGTTAGCATCCTCGATTCCTTCAA<br>ACGCGTCGACTCGGTCCAGGTCTTCATT	construct suicide plasmid pDM4_Δ*atpA*(A1+A2) for atpA knockout
atpF_A1_SacI<br>atpF_A1_R<br>atpF_A2_F<br>atpF_A2_SalI	GGAGAGCTCTCTGATTGCTGGTCTGTTG<br>AGCGACAAGTCCGAGGATTGTTGCGTTA<br>CAATCCTCGGACTTGTCGCTGAACTGTAA<br>ACGCGTCGACATGATAACGCCTGCCATTA	construct suicide plasmid pDM4_Δ*atpF*(A1+A2) for atpA knockout
dsbA_A1_SacI<br>dsbA_A1_R<br>dsbA_A2_F<br>dsbA_A2_Sal	GGAGAGCTCGCGACAGATGAGCTGATT<br>CAGCATACTGCTCTCCGATTAATACATAGGTG<br>AATCGGAGAGCAGTATGCTGATACAGTGAA<br>ACGCGTCGACTGCTCAACATCCACATCA	construct suicide plasmid pDM4_Δ*dsbA*(A1+A2) for dsbA knockout

dsbB_A1_SacI<br>dsbB_A1_R<br>dsbB_A2_F<br>dsbB_A2_SalI	GGAGAGCTCGGTCTGCTGTCATCCATT<br>TAAGCGATAAGCCTTGTGAACATTGGTT<br>TTCACAAGGCTTATCGCTTACCTGATTGTC<br>ACGCGTCGACATATCATTAACGCTGCTACG	construct suicide plasmid pDM4_Δ*dsbB*(A1+A2) for dsbB knockout
ompF_A1_SacI<br>ompF_A1_R<br>ompF_A2_F<br>ompF_A2_SalI	GGAGAGCTCTGGCATTCTGGATGTCTG<br>ATTAGAACTGCAGTACCTGCTACTAACAGA<br>GCAGGTACTGCAGTTCTAATAGCACACCTCT<br>ACGCGTCGACAACAGCAGTTCCTGAGTG	construct suicide plasmid pDM4_Δ*ompF*(A1+A2) for ompF knockout
dsbD_A1_SacI<br>dsbD_A1_R<br>dsbD_A2_F<br>dsbD_A2_SalI	GGAGAGCTCCGCCGACTAACATCCTTC<br>GTCGGTAGGCTTGAGCCATGAGAGGTAATC<br>CATGGCTCAAGCCTACCGACAATTCTCTT<br>ACGCGTCGACTCCGATAACGCCTGATAAC	construct suicide plasmid pDM4_Δ*dsbD* (A1+A2) for dsbD knockout
Pmic_-210_F<br>Pmic_-10_R	TGGAACGAGATGTACTGAA<br>CCCTATCGGTTGTTTGTTAT	amplify the promoter of mccPDI (Pmic) using PCR
Pmic_-210_F<br>Pmic_-35_R	TGGAACGAGATGTACTGAA<br>TGCGTTCAAGATAGCCAATA	amplify the fragment 1 of Pmic using PCR
Pmic_-210_F<br>Pmic_-61_R	TGGAACGAGATGTACTGAA<br>ATGTAATGAAAGTGAAATTGT	amplify the fragment 2 of Pmic using PCR
Pmic_-210_F<br>Pmic_-81_R	TGGAACGAGATGTACTGAA<br>TGTGATTAAATGTAAATATGTG	amplify the fragment 3 of Pmic using PCR
Pmic_-210_F<br>Pmic_-99_R	TGGAACGAGATGTACTGAA<br>TGTGAAAAAATTTGTATGT	amplify the fragment 4 of Pmic using PCR
Pmic_-210_F:<br>Pmic_-116_R	TGGAACGAGATGTACTGAA<br>GTTTTCGAGGTTGTGTAAT	amplify the fragment 5 of Pmic using PCR
Pmic_-134_F<br>Pmic_-10_R	ATTACACAACCTCGAAAAC<br>CCCTATCGGTTGTTTGTTAT	amplify the fragment 6 of Pmic using PCR
Pmic_-117_F<br>Pmic_-10_R	ACATACAAATTTTTTCACA<br>CCCTATCGGTTGTTTGTTAT	amplify the fragment 7 of Pmic using PCR
Pmic_-102_F<br>Pmic_-10_R	CACATATTTACATTTAATCACA<br>CCCTATCGGTTGTTTGTTAT	amplify the fragment 8 of Pmic using PCR
Pmic_-35/-84_F<br>Pmic_-35/-84_R	TCACACAATTTCACTTTCATTACATTTTTGTTATTGGCTATCTTGAACGCA<br>TGCGTTCAAGATAGCCAATAACAAAAATGTAATGAAAGTGAAATTGTGTGA	generate the fragment 9 of Pmic using PCR
Pmic_-61/-110_F<br>Pmic_-61/-110_R	AATTTTTTCACATATTTACATTTAATCACACAATTTCACTTTCATTACAT<br>ATGTAATGAAAGTGAAATTGTGTGATTAAATGTAAATATGTGAAAAAATT	generate the fragment 10 of Pmic using PCR
Pmic_-433_F<br>Pmic_-233_R	GGGAAACAGCGGTGAATA<br>GATGAAACCAGTTTACAGGAC	amplify DNA fragment located at position from -233 bp to -433bp relative to the start codon of mcpM gene (Pmic-233/-433)
PmicD_-163_F<br>PmicB_-20_R	CTATCCAATGCTGTTACATGC<br>ACCTTTCCGTCAACAAGAG	amplify DNA fragment located at position from -20 bp to -163 bp relative to the start codon of mcpD gene ($P_{micD\text{-}20/\text{-}163}$)
atpE-F<br>atpE-R	CCGGAATTCATGGAAAACCTGAATATGGA<br>CGCGTCGACCTAATGGTGATGGTGATGATGCGCGACAGCGAACATCACGTA	amplify atpE gene as control
pCR2.1_Pmic_F<br>pCR2.1_mcpM_SalI	TGGAACGAGATGTACTGAA<br>ACGCGTCGACTTAATGGTGATGGTGATGATGTCGGTTACATGTTCCGCCA	construct recombinant plasmid pCR::P*mic*+*mcpM* for mcpM expression under the endogenous promoter

qPCR_mcpA_F<br>qPCR_mcpA_R	AGCCGTTTATTCCCGCATGT<br>GCCTGATGCATCCGCAGTAT	Measure transcription of *mcpA* using quantitative PCR under different conditions and timepoints
qPCR_mcpB_F<br>qPCR_mcpB_R	AATGGATGGACGCTGCACAT<br>ATCAGCTGAATGCGGTCTGG	Measure transcription of *mcpB* using quantitative PCR under different conditions and timepoints
qPCR_mcpI_F<br>qPCR_mcpI_R	ATGAATCTTGCCGTGGAAAA<br>TGGAGGCGCTACTATGTTT	Measure transcription of *mcpI* using quantitative PCR under different conditions and timepoints
qPCR_mcpM_F<br>qPCR_mcpM_R	TAGGAATGGCAAGAGGTA<br>CTGAACAACTACTGGAACT	Measure transcription of *mcpM* using quantitative PCR under different conditions and timepoints
qPCR_rpoD_F<br>qPCR_rpoD_R	AGGTATCGCTGGTTTCGTTG<br>TTCGTACGCAAGAACGTCTG	Housekeeping gene for quantitative PCR experiments

Supplemental Table S3. Plasmids used in this study

Plasmids	Relevant characteristics	Reference
**pMMB207 vector (p207)**	Cm^r, RSF1010 derivative, *IncQ lacI*^{q} Tac *ori*T	Morales et al. 1991
p207::*envZ*	Cm^r, pMMB207 containing the *envZ* with a His.tag at the C-terminus	This study
p207::*envZ* H243A	Cm^r, pMMB207 containing the *envZ* H243A with a His.tag at the C-terminus	This study
p207::*ompR*	Cm^r, pMMB207 containing the *ompR* with a His.tag at the C-terminus	This study
p207::*mcpM*	Cm^r, pMMB207 containing the mcpM gene with a His.tag at the C-terminus	This study
p207::*mcpM* R5A	Cm^r, pMMB207 containing the mcpM R5A gene with a His.tag at the C-terminus	This study
p207::*mcpM* E11A	Cm^r, pMMB207 containing the mcpM E11A gene with a His.tag at the C-terminus	This study
p207::*mcpM* V15A	Cm^r, pMMB207 containing the mcpM V15A gene with a His.tag at the C-terminus	This study
p207::*mcpM* S16A	Cm^r, pMMB207 containing the mcpM S16A gene with a His.tag at the C-terminus	This study
p207::*mcpM* V15A/S16A	Cm^r, pMMB207 containing the mcpM V15A/S16A gene with a His.tag at the C-terminus	This study
p207::*mcpM* G17A	Cm^r, pMMB207 containing the mcpM G17A gene with a His.tag at the C-terminus	This study
p207::*mcpM* G18A	Cm^r, pMMB207 containing the mcpM G18A gene with a His.tag at the C-terminus	This study
p207::*mcpM* G17A/G18A	Cm^r, pMMB207 containing the mcpM G17A/G18A gene with a His.tag at the C-terminus	This study
p207::*mcpM* G17P	Cm^r, pMMB207 containing the mcpM G17P gene with a His.tag at the C-terminus	This study
p207::*mcpM* G18P	Cm^r, pMMB207 containing the mcpM G18P gene with a His.tag at the C-terminus	This study
p207::*mcpM* N19A	Cm^r, pMMB207 containing the mcpM N19A gene with a His.tag at the C-terminus	This study
p207::*mcpM* G26P	Cm^r, pMMB207 containing the mcpM G26P gene with a His.tag at the C-terminus	This study
p207::*mcpM* G27P	Cm^r, pMMB207 containing the mcpM G27P gene with a His.tag at the C-terminus	This study
p207::*mcpM* S33A	Cm^r, pMMB207 containing the mcpM S33A gene with a His.tag at the C-terminus	This study
p207::*mcpM* G35P	Cm^r, pMMB207 containing the mcpM G35P gene with a His.tag at the C-terminus	This study
p207::*mcpM* A36D	Cm^r, pMMB207 containing the mcpM A36D gene with a His.tag at the C-terminus	This study
p207::*mcpM* R37A	Cm^r, pMMB207 containing the mcpM R37A gene with a His.tag at the C-terminus	This study
p207::*mcpM* G41P	Cm^r, pMMB207 containing the mcpM G41P gene with a His.tag at the C-terminus	This study
p207::*mcpM* C57A	Cm^r, pMMB207 containing the mcpM C57A gene with a His.tag at the C-terminus	This study
p207::*mcpM* C90A	Cm^r, pMMB207 containing the mcpM C90A gene with a His.tag at the C-terminus	This study
p207::*mcpM* C109A	Cm^r, pMMB207 containing the mcpM C109A gene with a His.tag at the C-terminus	This study
p207::*mcpM* C118A	Cm^r, pMMB207 containing the mcpM C118A gene with a His.tag at the C-terminus	This study
p207::*mcpM* Δ1-18	Cm^r, pMMB207 containing the mutant mcpM (first leader peptide sequence 1-18 deleted) gene with a His.tag at the C-terminus	This study
p207::*mcpM* Δ1-36	Cm^r, pMMB207 containing the mutant mcpM (both leader peptide sequences 1-36 deleted) gene with a His.tag at the C-terminus	This study
p207::*mcpM* Δ19-36	Cm^r, pMMB207 containing the mutant mcpM (secondary leader peptide sequence 19-36 deleted) with a His.tag at the C-terminus	This study
p207::$ColV_{1-15}$+$mcpM_{\Delta 1-18}$	Cm^r, pMMB207 containing the recombinant mcpM (replacement of the first leader peptide sequence1-18 by colicin V leader sequence) with a His.tag at the C-terminus	This study
**pCR2.1-TOPO vector (pCR)**	Amp^r, cloning vector	Invitrogen
pCR::P*mic*+*mcpM*	Amp^r, pCR2.1 containing the mcpM gene with a His.tag at the C-terminus under the endogenous promoter control	This study
**pPAL7 vector (pPAL)**	Amp^r, a T7-based expression vector with an N-terminal Profinity eXact tag	Bio-Rad
pPAL7-*ompR*	Amp^r, pPAL7 containing the *ompR*	This study
pPAL7-*ompR* D55A	Amp^r, pPAL7 containing the *ompR* D55A	This study

pPAL7-*XRE*	Amp^r, pPAL7 containing the *XRE*	This study
**pDM4 vector**	Cm^r, Suicide vector with an R6K origin (pir-requiring) and *sac*BR of *Bacillus subtilis*	Milton et al.1996
pDM4-Δ*envZ*(A1+A2)	Cm^r, pDM4 containing the flanking region sequences of *envZ*	This study
pDM4-Δ*ompR*(A1+A2)	Cm^r, pDM4 containing the flanking region sequences of *ompR*	This study
pDM4-Δ*mcpM*(A1+A2)	Cm^r, pDM4 containing the flanking region sequences of *mcpM*	This study
pDM4-Δ*mcpB*(A1+A2)	Cm^r, pDM4 containing the flanking region sequences of *mcpB*	This study
pDM4-Δ*mcpD*(A1+A2)	Cm^r, pDM4 containing the flanking region sequences of *mcpD*	This study
pDM4-Δ*dsbA*(A1+A2)	Cm^r, pDM4 containing the flanking region sequences of *dsbA*	This study
pDM4-Δ*dsbB*(A1+A2)	Cm^r, pDM4 containing the flanking region sequences of *dsbB*	This study
pDM4-Δ*dsbD*(A1+A2)	Cm^r, pDM4 containing the flanking region sequences of *dsbD*	This study

References:

Sawant AA, Casavant NC, Call DR, Besser TE. 2011. Proximity-dependent inhibition in *Escherichia coli* isolates from cattle. Appl Environ Microbiol **77:**2345-2351.

Baba T, Ara T, Hasegawa M, Takai Y, Okumura Y, Baba M, Datsenko KA, Tomita M, Wanner BL, Mori H. 2006. Construction of Escherichia coli K-12 in-frame, single-gene knockout mutants: the Keio collection. Mol Syst Biol. **2:** 2006.0008.

Eberhart LJ, Ochoa JN, Besser TE, Call DR. 2014. Microcin MccPDI reduces the prevalence of susceptible Escherichia coli in neonatal calves. J Appl Microbiol. **117(2)**:340-6.

Eberhart LJ, Deringer JR, Brayton KA, Sawant AA, Besser TE, Call DR. 2012. Characterization of a novel microcin that kills enterohemorrhagic *Escherichia coli* O157:H7 and O26. Appl Environ Microbiol **78**:6592-6599.

Zhao Z, Eberhart LJ, Orfe LH, Lu SY, Besser TE, Call DR. 2015. Genome-Wide Screening Identifies Six Genes That Are Associated with Susceptibility to Escherichia coli Microcin PDI. Appl Environ Microbiol **81:**6953-6963.

Simon R, Priefer U, Pühler A. 1983. A Broad Host Range Mobilization System for *In Vivo* Genetic Engineering: Transposon Mutagenesis in Gram Negative Bacteria. Nat Biotechnol **1**:784–791.

Morales VM, Backman A, Bagdasarian M. 1991. A series of wide-host-range low-copy-number vectors that allow direct screening for recombinants. Gene **97**: 39–47.

Valdivia RH, Falkow S.1996. Bacterial genetics by flow cytometry: rapid isolation of Salmonella typhimurium acid-inducible promoters by differential fluorescence induction. Mol Microbiol. **22(2)**:367-78.

Milton DL, O'Toole R, Horstedt P, Wolf-Watz H. 1996. Flagellin A is essential for the virulence of Vibrio anguillarum. J Bacteriol **178**: 1310–1319.

Bland DM, Eisele NA, Keleher LL, Anderson PE, Anderson DM. 2011. Novel genetic tools for diaminopimelic acid selection in virulence studies of Yersinia pestis. PLoS One. **6(3)**:e17352.
